# Topoisomerases I and III inhibit R-loop formation to prevent unregulated replication in the chromosomal Ter region of *Escherichia coli*

**DOI:** 10.1371/journal.pgen.1007668

**Published:** 2018-09-17

**Authors:** Julien Brochu, Émilie Vlachos-Breton, Sarah Sutherland, Makisha Martel, Marc Drolet

**Affiliations:** Département de microbiologie, infectiologie et immunologie, Université de Montréal, Succ. Centre-ville, Montréal, PQ, Canada; Duke University, UNITED STATES

## Abstract

Type 1A topoisomerases (topos) are the only ubiquitous topos. *E*. *coli* has two type 1A topos, topo I (*topA*) and topo III (*topB*). Topo I relaxes negative supercoiling in part to inhibit R-loop formation. To grow, *topA* mutants acquire compensatory mutations, base substitutions in *gyrA* or *gyrB* (gyrase) or amplifications of a DNA region including *parC* and *parE* (topo IV). *topB* mutants grow normally and topo III binds tightly to single-stranded DNA. What functions topo I and III share *in vivo* and how cells lacking these important enzymes can survive is unclear. Previously, a *gyrB*(Ts) compensatory mutation was used to construct *topA topB* null mutants. These mutants form very long filaments and accumulate diffuse DNA, phenotypes that appears to be related to replication from R-loops. Here, next generation sequencing and qPCR for marker frequency analysis were used to further define the functions of type 1A topos. The results reveal the presence of a RNase HI-sensitive origin of replication in the terminus (Ter) region of the chromosome that is more active in *topA topB* cells than in *topA* and *rnhA* (RNase HI) null cells. The S9.6 antibodies specific to DNA:RNA hybrids were used in dot-blot experiments to show the accumulation of R-loops in *rnhA*, *topA* and *topA topB* null cells. Moreover *topA topB gyrB*(Ts) strains, but not a *topA gyrB*(Ts) strain, were found to carry a *parC parE* amplification. When a *topA gyrB*(Ts) mutant carried a plasmid producing topo IV, *topB* null transductants did not have *parC parE* amplifications. Altogether, the data indicate that in *E*. *coli* type 1A topos are required to inhibit R-loop formation/accumulation mostly to prevent unregulated replication in Ter, and that they are essential to prevent excess negative supercoiling and its detrimental effects on cell growth and survival.

## Author summary

DNA topoisomerases are nicking closing enzymes with strand passage activity that solves the topological problems inherent to the double-helical structure of DNA. Topos of the type 1A family are the only ubiquitous topos. They are classified in two subfamilies, topo I and topo III respectively found in bacteria only and in organisms from the three domains of life. The prototype enzymes of these two subfamilies are topo I and topo III from *Escherichia coli*. Recent data suggest that duplications leading to topo I and III subfamilies occurred in the Last Common Universal Ancestor of the three domains of life. In this context, our finding reported here that both *E*. *coli* topo I and III control R-loop formation/accumulation, mostly to inhibit unregulated replication, may suggest that R-loops have been a problem early in the evolution of life. Furthermore, our data show that *E*. *coli* cells can survive in the absence of type 1A topos, owing to the surproduction of topo IV that can relax excess negative supercoiling and prevent R-loop formation. Thus, our results strongly suggest that a major function of type 1A topos is to control R-loop formation to preserve the integrity of the genome.

## Introduction

DNA topoisomerases (topos) are nicking-closing enzymes that solve the topological problems inherent to the double-helical structure of the DNA [1]. Such problems arise during DNA transactions including replication, transcription and recombination and must be solved in order for these processes to be completed, and to maintain the integrity of the genome. Type I topos cut one DNA strand and either use a strand passage (1A) or a rotation mechanism (1B) to alter the topology of DNA. Type II enzymes cut two DNA strands and use a strand passage mechanism to change the topology of DNA.

Type 1A topos are the sole ubiquitous topos, being present in the three domains of life [2, 3]. They require single-stranded DNA (ssDNA) for binding, a substrate that is mostly provided by negatively supercoiled DNA for some of these enzymes, as is the case for bacterial topos I, the members of the first type 1A topo subfamily, encoded by *topA* [1]. The prototype enzyme of this group, also the first topo to be discovered, is topo I from *E*. *coli* [4]. To form visible colonies on solid media, *E*. *coli topA* null mutants need to acquire compensatory mutations in *gyrA* or *gyrB*, reducing the supercoiling activity of DNA gyrase [5, 6], or amplifications of a chromosomal DNA region carrying *parC* and *parE* [7, 8], the two subunits of topo IV (a type II topo), increasing the cellular DNA relaxation activity. In fact, topo IV, at its wild-type cellular level, does play a role in the regulation of supercoiling in *E*. *coli* [9]. These observations lead to a model in which the chromosomal negative supercoiling in the cell is set by the opposing enzymatic activities of DNA gyrase, introducing negative supercoiling, and topo I and IV relaxing it [9]. In fact, the major function of topo I in supercoiling regulation, is the relaxation of transcription-induced negative supercoiling behind moving RNA polymerases (RNAPs) [10]. Topo I physically interacts with RNAP in *E*. *coli* [11, 12] and this interaction is also found between *M*. *tuberculosis* topo I and RNAP, though in that case by a distinct mechanism [13].

In *E*. *coli*, one major consequence of transcription-induced negative supercoiling if not relaxed, e.g. in *topA* null mutants, is R-loop formation [14]. An R-loop forms when the nascent RNA re-anneals with the template strand behind the moving RNAP [14]. Overproducing RNase HI (*rnhA*), the enzyme degrading the RNA of an R-loop, significantly rescue the growth defect of *topA* null mutants [15], and double *topA rnhA* null mutants do not grow despite the presence of a *gyrB* compensatory mutation [15, 16]. Evidence for R-loop formation inhibiting transcription, causing RNA degradation and hypernegative supercoiling in *topA* null mutants has been presented [17]. Furthermore, R-loop formation in yeast and human cells has been reported [18–21]. It is now clear that R-loops can have a wide range of positive and negative effects on cell physiology [22, 23].

A second group of type 1A topos, the topo III subfamily, is widely distributed in the three domains of life [2, 3]. Topo III is characterized by a stronger requirement for ssDNA as compared to topo I [1]. The first enzyme of this subfamily has been found in *E*. *coli* and is encoded by *topB* [24, 25]. *In vitro*, *E*.*coli* topo III has a strong decatenase activity but a poor relaxation activity on DNA with a wild-type supercoiling density at 37°C [24]. In fact, topo III plays no role in global supercoiling regulation in *E*. *coli* [9, 26]. As opposed to *topA* mutants, *topB* mutants grow normally, do not accumulate compensatory mutations and display no obvious phenotypes [25]. *In vitro* topo III can act as a decatenase to fully support replication of a circular dsDNA template [27]. Evidence for topo III acting as a replicative decatenase, as a back-up for the main cellular decatenase topo IV has been presented [28]. *In vitro*, an R-looped DNA template strongly stimulates the relaxation activity of topo III [29].

The initial studies performed with double *topA topB* null mutants of *E*. *coli*, lead to the conclusion that both topo I and III share a unique and essential function [30]. *topA* null cells carrying a gyrase compensatory mutation and depleted of topo III activity generated very long filaments fully packed with unsegregated and diffuse DNA, and eventually stopped growing [30]. These phenotypes were not observed for single type 1A topo mutants. Additionally, cells lacking both type 1A topos were said to be non-viable as *topB* null transductants of a *topA* null strain could not be obtained after an overnight incubation on minimal medium. As these phenotypes could be corrected by deleting *recA*, it was also concluded that they were related to homologous recombination [30].

In later studies, *topA topB* null transductants in strains carrying a gyrase compensatory mutation could be obtained after 48 hours of incubation [31, 32]. These results demonstrated that *E*. *coli* cells can survive without type 1A topos but did not show if compensatory mutations were required for viability, and therefore how such mutants could survive. The fact that the typical phenotypes of cells lacking type 1A topos were observed, were exacerbated by decreasing the incubation temperature in strain carrying a *gyrB*(Ts) compensatory mutation, and were largely corrected by overproducing RNase HI, suggested that deleting *topB* in *topA* null mutants exacerbated the supercoiling and R-loop-dependent phenotypes of *topA* null cells [33]. More recently, replication from R-loops was shown to be activated in *topA topB* null cells [34].

Replication from R-loops was first described in *E*. *coli* cells lacking RNase HI (*rnhA*) [35, 36]. This replication was named “constitutive stable DNA replication” (cSDR) because, as opposed to the normal one from the chromosomal origin *oriC* (requiring *de novo* DnaA synthesis), it could go on for several hours after the full inhibition of protein synthesis. Replication from R-loops is initiated via the PriA-dependent primosome that also includes PriB and DnaT proteins [36]. The origins of replication from R-loops were tentatively mapped and were named *oriKs* [37]. The RecA recombinase has been shown to be involved in the initiation step of cSDR [38], possibly at the step of R-loop formation since this protein is able to promote R-loop formation *in vitro* [39, 40]. Since the strong phenotypes of *topA topB* null cells were significantly corrected by a *dnaT* mutation, it was concluded that cSDR was largely responsible for the sickness of cells lacking type 1A topos [33]. Furthermore, the observation that deleting *recA* corrected the strong phenotypes of *topA topB* null cells was explained, at least in part, in the context of the role of RecA in cSDR [33]. This is also supported by the observation that the positive effects of RNase H overproduction on the growth and phenotypes of *topA topB* null cells is not seen when *recA* is deleted [33].

Many things are still unclear regarding the phenotypes of *topA topB* null cells and the role of type 1A topos. For example, topo III is a protein of low abundance and there is no evidence that, at least at this level, it could act on R-loops to inhibit cSDR. In fact, no cSDR is detected in cells lacking topo III activity and deleting *topB* has no effect on growth, cell morphology and cSDR in *rnhA* mutant [34]. Furthermore, it is not obvious why a high level of cSDR could generate the severe phenotypes of *topA topB* null mutants, especially when considering the fact that these phenotypes are not observed in *rnhA* mutants that have an even higher level of cSDR [34]. In this work, we have used next-generation sequencing (NGS) and qPCR for marker frequency analysis (MFA) to further characterize the roles of type 1A topos. Moreover, we have used the DNA:RNA hybrids specific antibody S9.6 [41] to detect R-loops in *rnhA*, *topA* and *topA topB* null cells. Our results reveal three important findings: 1- Type 1A topos from both subfamilies can inhibit R-loop formation/accumulation mostly to prevent unregulated replication from R-loops. 2- High levels of replication from R-loops in the terminus region of the circular chromosome appear to be largely responsible for the strong phenotypes of *topA topB* null mutants. 3- *topA topB* null mutants are able to grow owing to the amplification of a chromosomal DNA region carrying the *parC* and *parE* genes that leads to topo IV overproduction.

## Results

### Effect of a *dnaT* mutation and a DNA inversion on the putative *oriK* in the Ter region of *rnhA* null cells

**Fig 1:**
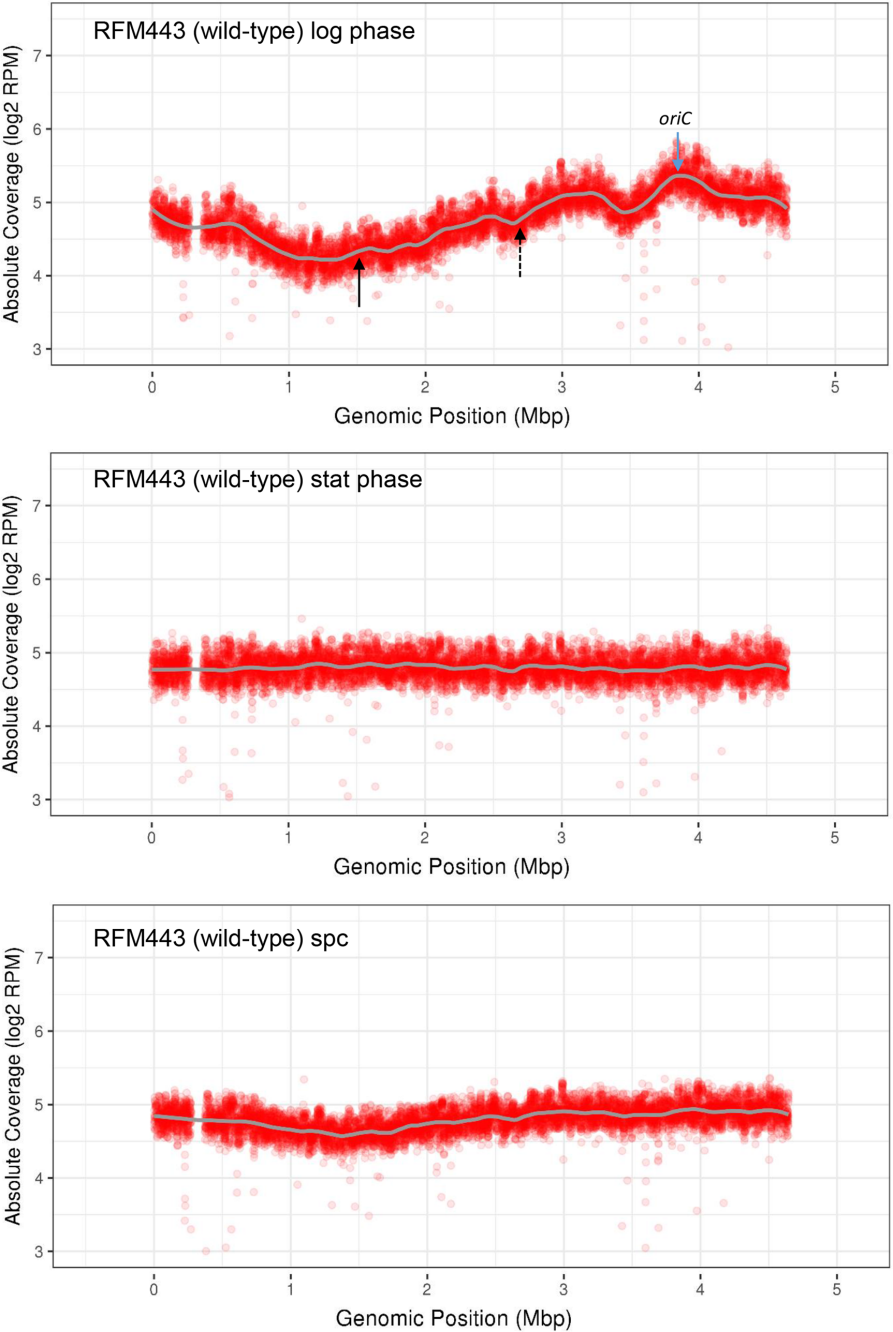
Replication profiles of the wild-type strain RFM443 under various growth conditions. Wild-type cells (RFM443) were grown at 37°C to log phase (top panel), stationary phase (middle panel) or log phase followed by a spectinomycin (spc) treatment (bottom panel), and genomic DNA was extracted for NGS as described in Materials and Methods. The absolute read counts (Log2) were plotted against chromosomal coordinates (W3110). The gray line is the loess regression curve (see Materials and Methods). The blue arrow points to the origin of replication (*oriC*) and the full and dashed black arrows respectively point to *ydcM* and *lepA* genes. The gap at position around 0.3 corresponds to the *Δ(codB-lacI)3* deletion carried by the strains used in this work (see S1 Table).

We used MFA by NGS to map the putative *oriKs* on the chromosome of our isogenic strains. This approach was recently used for *rnhA* null strains, and five to ten peaks that could correspond to sites of replication initiation from R-loops were identified, including one prominent peak in the Ter region [42, 43]. This approach is also very useful for the detection of various DNA rearrangements such as duplications/amplifications and inversions [44]. For our experiments, genomic DNA was extracted from log phase cells (DO_600_, 0.4) treated for two hours with spectinomycin (400 μg/ml), a protein synthesis inhibitor. With this treatment, we have previously shown that replication was fully completed in wild-type cells, whereas it could still be detected (cSDR) in *rnhA*, *topA* and *topA topB* null cells, unless RNase HI was overproduced for the type 1A topos mutants [34]. Fig 1 shows the results with the wild-type strain not treated (log phase cells, top panel and stationary phase cells, middle panel) or treated with spectinomycin (log phase cells, bottom panel). For log phase cells (top panel), a typical wild-type replication profile is seen where the highest and lowest copy numbers respectively are found in the *oriC* and Ter regions. This is the expected result for bidirectional replication initiated at *oriC* and terminated following the merging of the two replication forks in the Ter region. For stationary phase cells (middle panel), a flattened profile with no significant peaks is generated, also as expected since *oriC*-dependent replication is inhibited in non-growing stationary phase cells. A uniform profile is also detected for wild-type cells treated with spectinomycin for two hours (bottom panel). This is in agreement with our previous results showing that *oriC*-dependent replication was completed and cSDR not activated in wild-type cells treated with spectinomycin for two hours [34]. Thus MFA by NGS reveals that replication from *oriC* is active in wild-type log phase cells but not in stationary phase cells or in cells treated with spectinomycin for 2 hours.

**Fig 2:**
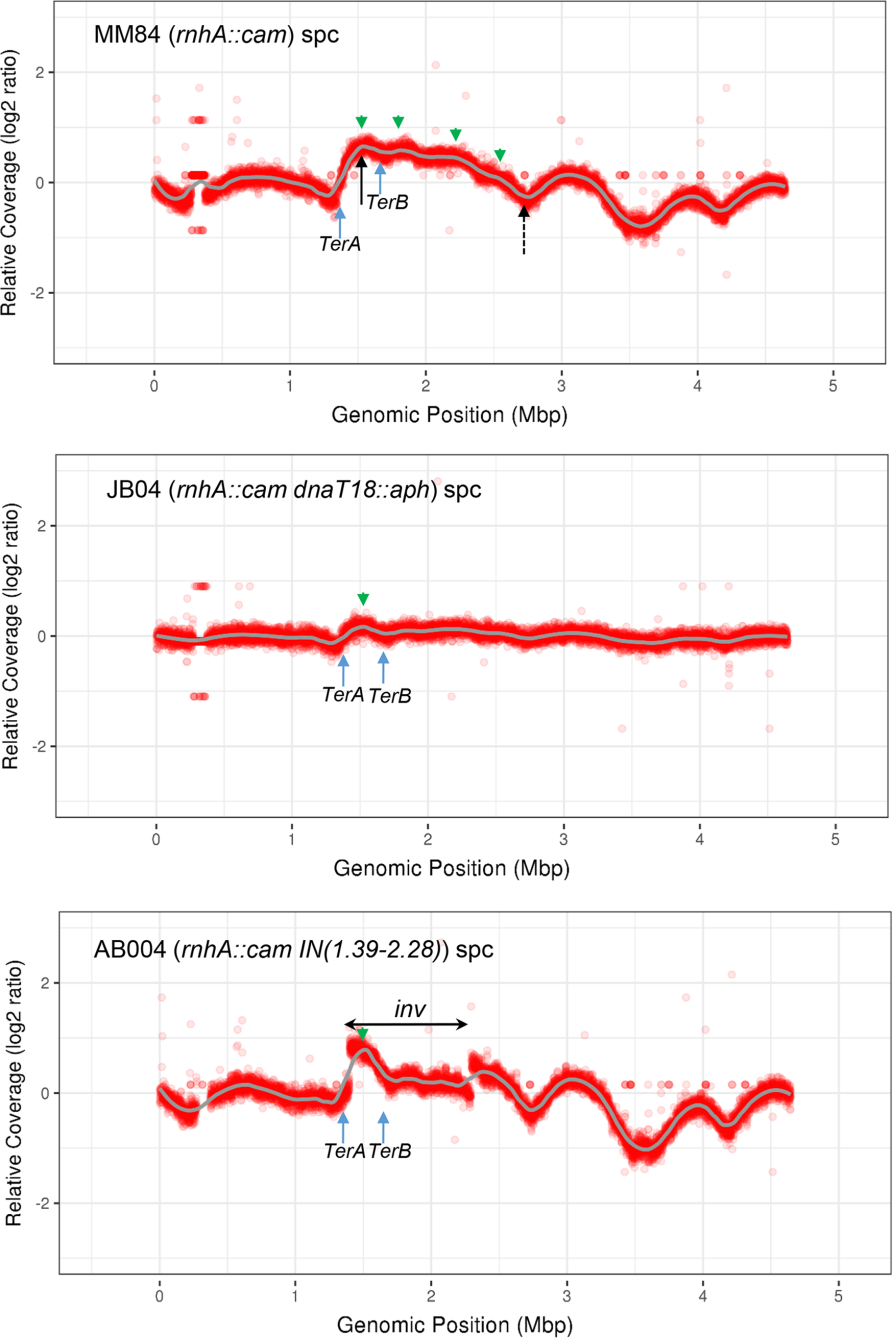
Replication profiles of *rnhA* null strains. *rnhA*::*cam* (MM84), *rnhA*::*cam dnaT18*::*aph* (JB04) and *rnhA*::*cam IN(1*.*39–2*.*28)* (AB004) cells were grown at 37°C to log phase and treated with spectinomycin (spc), and genomic DNA was extracted for NGS as described in Materials and Methods. The read counts (Log2) normalized against a wild-type (RFM443) spectinomycin treated control were plotted against chromosomal coordinates (W3110). The gray line is the loess regression curve (see Materials and Methods). The green arrows on the top of the profiles point to potential cSDR origins (*oriKs*) and the blue ones at the bottom of the profiles point to *TerA* and *TerB* polar replication termination sequences. The full and dashed black arrows in the top panel respectively point to *ydcM* and *lepA* genes. In the bottom panel, *inv* shows the chromosomal DNA inversion (1.39–2.28) in strain AB004.

Fig 2, top panel, shows that the treatment of *rnhA* null cells with spectinomycin generated a replication profile that is much more complex as compared to wild-type cells, with important fluctuations in copy numbers throughout the profile. In our cSDR study, we focused on a genomic region roughly delineated by the full and dashed black arrows (e.g. Fig 2 top panel) that respectively point to *ydcM* (left; genomic position 1.505) and *lepA* (right; genomic position 2.705) genes. These genes were used in our qPCR experiments and their position roughly correspond to the highest (*ydcM*) and lowest (*lepA*) copy numbers observed for *topA topB* null strains (see below; excepted *amp*). Green arrows in Fig 2, top panel, point to potential *oriK* sites (peaks or bumps). The left one at position 1.52 is flanked by the *TerA* and *TerB* sites and the others ones are found at positions 1.83, 2.23 and 2.54. A prominent peak at position 1.52 was previously mapped for *rnhA* null cells treated with chloramphenicol [42] that also inhibits protein synthesis and allow cSDR to be detected. Bumps around 1.83, 2.23 and 2.54 were also detected in previous studies [42, 43]. The profile suggests that replication initiated in the Ter region (peak at 1.52) is bidirectional and that the left- (counterclockwise) and right- (clockwise) moving forks are arrested respectively at *TerA* and *TerB*. In *E*. *coli*, replication forks are trapped within the Ter region by the Tus protein that binds to polar *Ter* sequences (*TerA* to *TerJ*). *Ter* sequences within the left portion of the chromosome block clockwise-moving forks, whereas those in the right portion of the chromosome block counterclockwise-moving forks [45]. Note also that for the *rnhA* null cells (Fig 2, top panel), the profile is asymmetric is this area with the drop in copy numbers being much more important at *TerA* than *TerB*. This can be explained by the presence of counterclockwise-moving replication forks from putative *oriKs* at positions 1.83, 2.23 and 2.54 that pass through the *TerB* site and are arrested at the *TerA* site.

**Fig 3:**
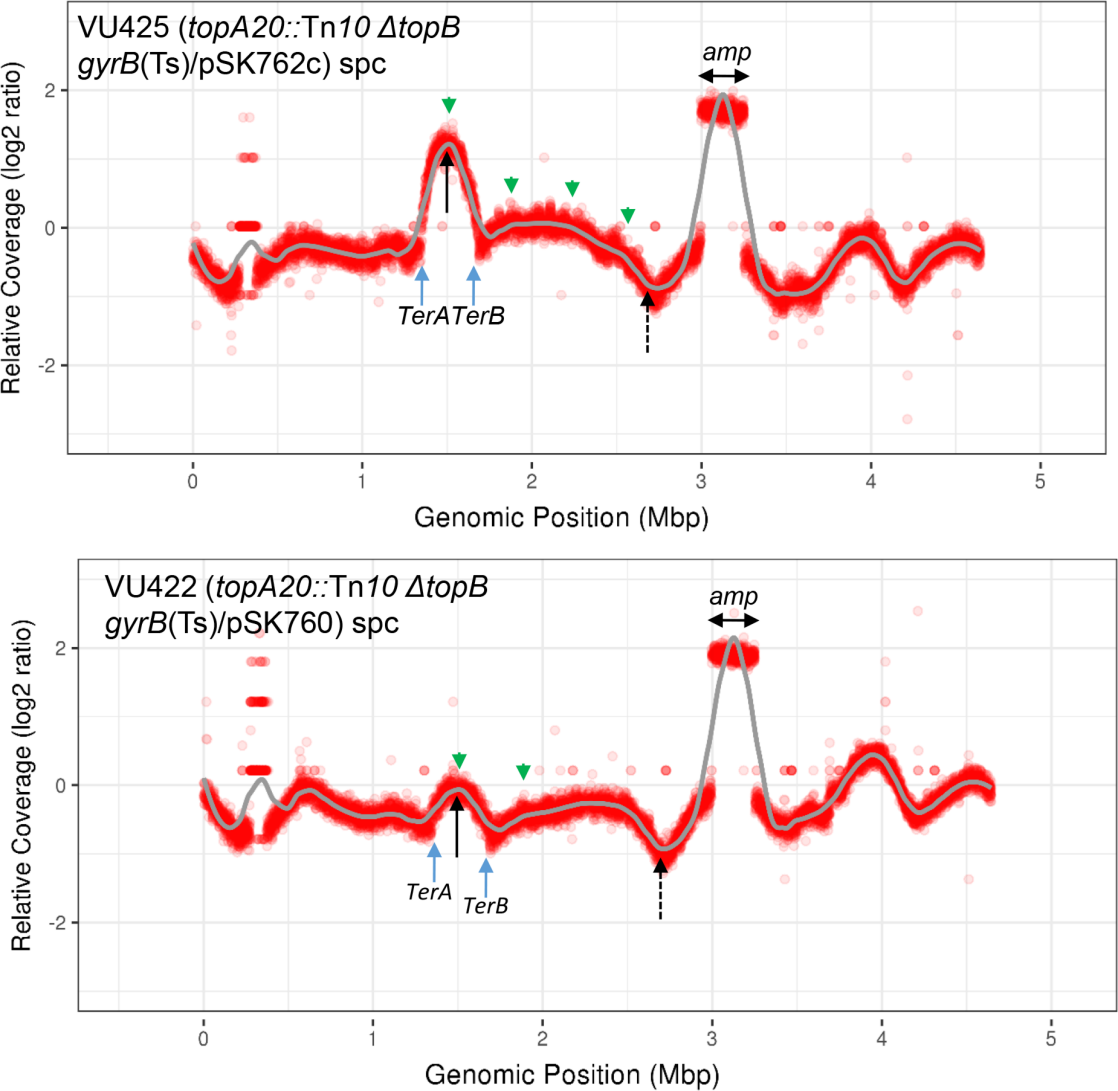
Replication profiles of *topA topB* null strains. *topA20*::Tn*10 ΔtopB gyrB*(Ts)/pSK762c (VU425), *topA20*::Tn*10 ΔtopB gyrB*(Ts)/pSK760 (VU422) cells were grown at 30°C to log phase and treated with spectinomycin (spc), and genomic DNA was extracted for NGS as described in Materials and Methods. The read counts (Log2) normalized against wild-type (RFM443) spectinomycin treated cells were plotted against chromosomal coordinates (W3110). The gray line is the loess regression curve (see Materials and Methods). The green arrows on the top of the profiles point to potential cSDR origins (*oriKs*) and the blue ones at the bottom of the profiles point to *TerA* and *TerB* polar replication termination sequences. The full and dashed black arrows respectively point to *ydcM* and *lepA* genes. *amp* shows the chromosomal DNA amplification (2.99–3.24) in strains VU422 and VU425.

MFA by NGS can also reveal the occurrence of collisions between replication and transcription. Such collisions, especially head-on as compared to co-directional, can have a major impact on cell physiology, mostly when they involve the heavily transcribed *rrn* (rRNA) operons [43, 46]. The impact of collisions with *rrn* operons are normally minimized because they are transcribed in the same orientation as the bi-directional replication forks coming from *oriC*. However, when cSDR is activated from *oriKs*, head-on collisions between replication forks and transcribed *rrn* operons are inevitable. As shown in Fig 2 top panel, such collisions can be revealed by the presence of “hollows” or “steps” at genomic positions corresponding to *rrnG* (2.73), the *rrnDBAC* cluster (3.42 to 3.69) and *rrnE* (4.21). Similar patterns were also observed for the replication profiles of *topA topB* null strains (Fig 3).

To further support the notion that the identified peaks or bumps correspond to *oriK* sites, we used a *dnaT18*::*aph* mutation that was previously isolated as a suppressor of the growth defect of a *topA rnhA* null mutant [47]. This mutation was shown to abolish cSDR in *rnhA* null mutants [33, 34]. This allele does not behave like a loss-of-function mutation because, as opposed to a *dnaT* null mutation that confers severe growth defects similar to *priA* null mutations [48], our *dnaT18*::*aph* mutation does not affect cell growth [33]. In fact, the *aph* cassette is inserted within the *dnaT* promoter region [33] and the results of our qRT-PCR experiments reveal that this insertion caused a 5 to 6-fold reduction in *dnaT* mRNA levels (S1 Fig). Importantly, we found that the expression of the *dnaC* gene that is believed to be co-transcribed with *dnaT* (*dnaTC* operon) [49] is not affected by the *dnaT18*::*aph* mutation (S1 Fig). Thus, the *dnaT18*::*aph* mutation is well-suited for the study of cSDR. Fig 2, middle panel, shows the replication profile of *dnaT18*::*aph rnhA* null cells treated with spectinomycin. It can be seen that the presence of this *dnaT* allele almost fully eliminated the peaks or bumps found in *rnhA* null cells, thus strongly suggesting that they indeed correspond to *oriK* sites.

Fig 2, bottom panel, shows the replication profile of an *rnhA* null strain (treated with spectinomycin) carrying a large DNA inversion (*inv*; from 1.39 to 2.28) in the Ter region that includes the *oriK* site at 1.52. We found that this strain has reduced cSDR activity as compared to the non-inverted *rnhA* null strain [47]. If the peak at 1.52 really corresponds to an origin of replication (a fixed origin of replication) its location should be modified accordingly in the strain carrying the inversion. This is what we found, i.e., the peak is still located at position 1.52 according to the W3110 reference strain. Thus, this peak is not the result of replication initiation triggered by the collision of bidirectional forks from *oriC*, as suggested to explain over-replication in the Ter region of *recG* cells [50]. Altogether, our results strongly suggest that the peak at position 1.52 in the Ter region of *rnhA* null cells corresponds to a fixed origin of replication that is R-loop- and PriA-primosome-dependent (*oriK*).

### The Ter-located *oriK* site is strongly activated in cells lacking type 1A topoisomerases

Fig 3 top panel shows the replication profile of *topA topB* null cells (all the *topA topB* null cells used in the present study also carry a *gyrB*(Ts) compensatory mutation) not overproducing RNase HI (strain VU425, *topA topB gyrB*(Ts)/pSK762c) and treated with spectinomycin. A prominent peak in the Ter region that corresponds to the *oriK* site mapped in *rnhA* null cells (genomic position 1.52) is detected, together with much smaller bumps at genomic positions 1.88, 2.23 and 2.56 (green arrows). When compared to the replication profile of *rnhA* null cells (Fig 2 top panel), it is obvious that the Ter-located *oriK* is significantly more active in the *topA topB* null mutant, whereas the *oriKs* lying outside Ter are much less active. As expected if these peaks and bumps correspond to *oriKs*, RNase HI overproduction (strain VU422, *topA topB gyrB*(Ts)/pSK760) considerably reduce their intensity; the small bumps outside Ter are no longer seen and the peak within the Ter region is much lower (Fig 3 bottom panel). cSDR is also activated in single *topA* null mutants, though its level is lower as compared to *topA topB* null cells. Indeed, S2 Fig shows the replication profile of single *topA* null cells treated with spectinomycin that demonstrates the low level of cSDR activity in this strain, with only one small peak being found in the Ter region (position 1.52). Thus deleting the *topB* gene in a *topA* null strain significantly increases cSDR activity in the Ter region. This result shows that topo III also acts at the level of cSDR initiation.

To further support our results of MFA by NGS we performed qPCR. As stated above we used probes that correspond to *ydcM* (genomic position 1.505) and *lepA* (genomic position 2.705). Their position roughly correspond to the highest (*ydcM*; excepted *amp*, see below) and lowest (*lepA*) copy numbers observed for *topA topB* null strains (Fig 3, top panel, full and dashed black arrows respectively). The histogram in S3 Fig shows the result of qPCR experiments with samples of the genomic DNA preps that were used for MFA by NGS. Columns with an error bar correspond to the qPCR results (at least two independent experiments) represented as the *ydcM*/*lepA* ratio. The ratios were also calculated from the results of MFA by NGS (columns with no error bars). It can be seen that very similar *ydcM*/*lepA* ratios are obtained with the two approaches, which confirms the results of MFA by NGS and validates the qPCR method.

**Fig 4:**
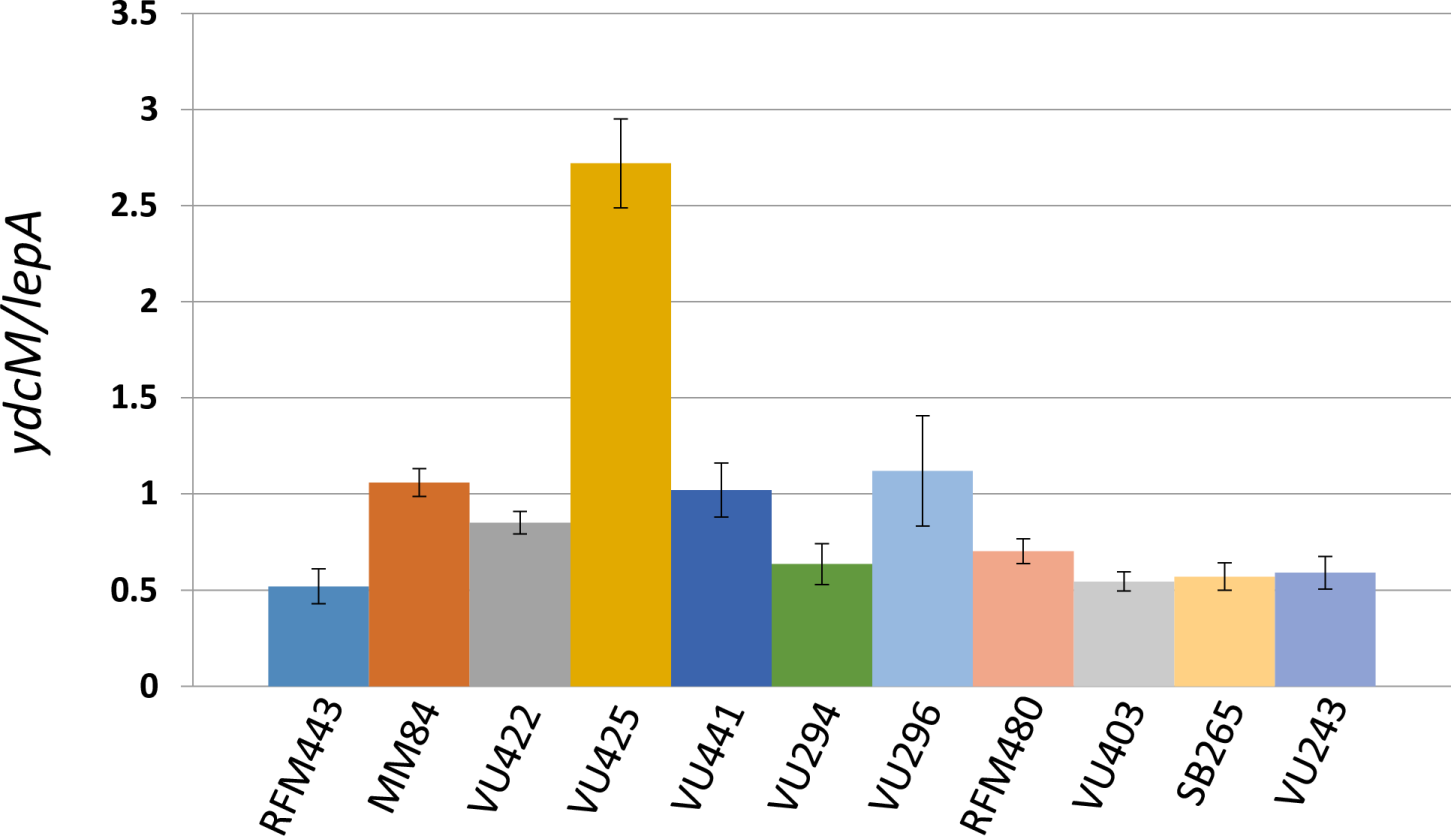
*ydcM/lepA* ratio in *E*. *coli rnhA*, *topA* and *topA topB* null mutants. Wild-type (RFM443) and *rnhA*::*cam* (MM84) cells were grown at 37°C to log phase, whereas *topA20*::Tn*10 ΔtopB gyrB*(Ts)/pSK760 (VU422), *topA20*::Tn*10 ΔtopB gyrB*(Ts)/pSK762c (VU425), *topA20*::Tn*10 ΔtopB dnaT18*::*aph gyrB*(Ts) (VU441), *Δ(topA cysB) gyrB*(Ts)/pEM001 (VU294), *Δ(topA cysB) gyrB*(Ts)/pEM003 (VU296), *topA20*::Tn*10 gyrB*(Ts) (RFM480), *ΔtopB761*::*kan gyrB*(Ts) (VU403), *Δ(topA cysB) gyrB*(Ts) *ΔrecA306 srlR301*::Tn*10* (SB265) and *Δ(topA cysB) ΔtopB*::*kan gyrB*(Ts) *ΔrecA306 srlR301*::Tn*10* (VU243) were grown at 30°C to log phase. Genomic DNA was extracted and qPCR was performed as described in Materials and Methods.

Fig 4 shows the *ydcM*/*lepA* ratios calculated from the results of qPCR experiments with genomic DNA samples from log phase cells not treated with spectinomycin. As it is the case for the DNA samples from cells treated with spectinomycin, the highest ratio is found for the *topA topB* null mutant not overproducing RNase HI (VU425, pSK762c no overproduction vs VU422, pSK760 overproduction). As expected, the ratio is much lower when the *dnaT18*::*aph* mutation is present in the *topA topB* null mutant (VU441). Importantly, while the ratio is significantly higher in the *topA* null mutant not overproducing RNase HI (VU296) as compared to the wild-type strain (RFM443), it is much higher when *topB* is also absent (VU425). This confirms the important regulatory role of topo III on cSDR initiation in the Ter region. However, this role is only observed when *topA* is also lacking as no increase in the *ydcM*/*lepA* ratio is observed for the isogenic *topB* null mutant (VU403) as compared to the wild-type strain. This result is in agreement with our previous finding that cSDR was not activated in a single *topB* null mutant [34]. Moreover, deleting *recA* in both *topA* (SB265) and *topA topB* (VU243) null mutants, restored the *ydcM*/*lepA* ratio to the level seen in wild-type cells. This is the expected result as RecA is required for the initiation step of cSDR [38]. Overall, when the ratios in Fig 4 (no spectinomycin) are compared to those shown in S3 Fig (spectinomycin) the differences between the strains are very similar but the ratios are lower. This is expected as in log phase cells not treated with spectinomycin, replication from *oriC* is active.

### Dot-blots with S9.6 antibodies reveal the accumulation of R-loops in cells lacking type 1A topoisomerases

**Fig 5:**
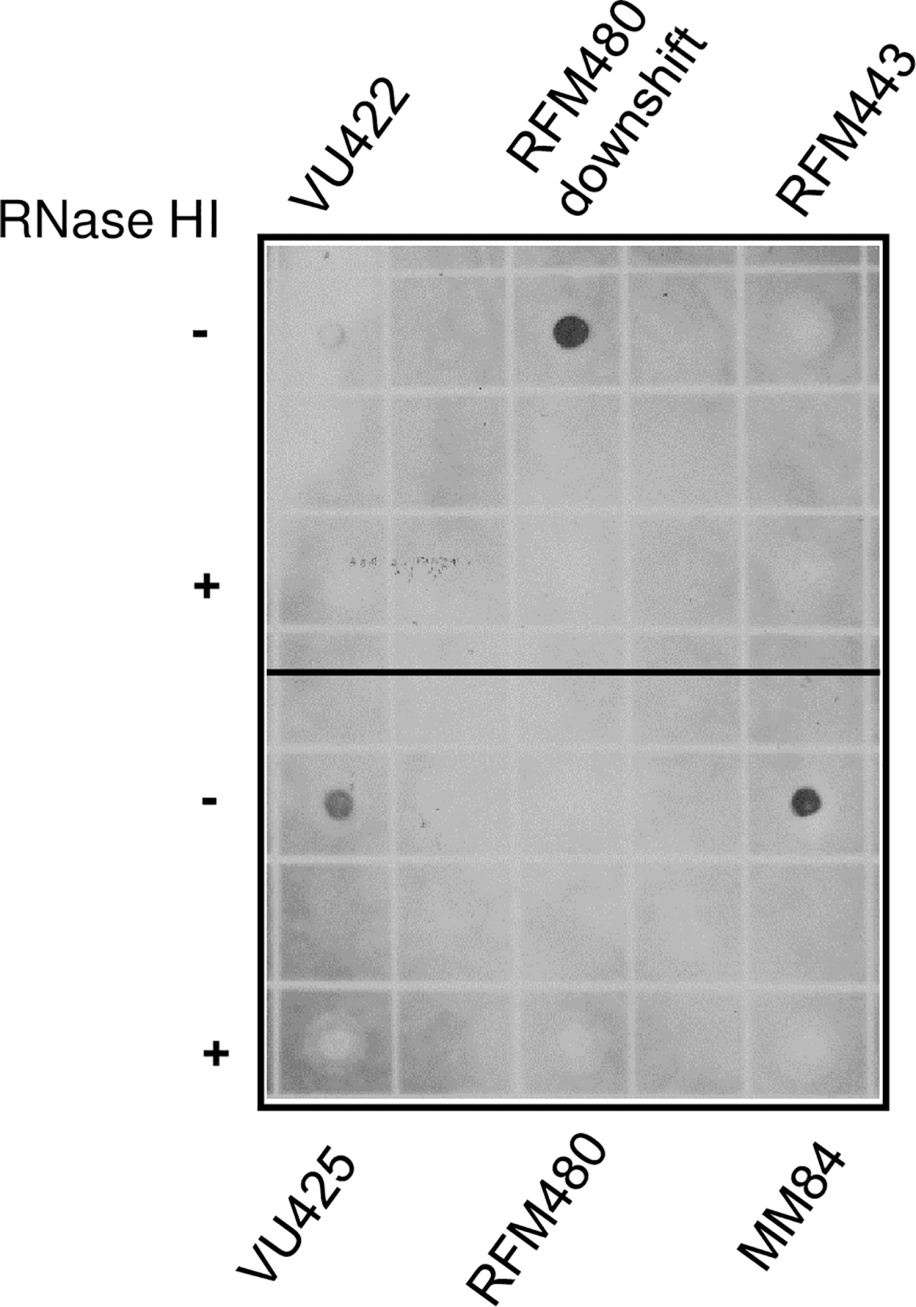
Dot blots with S9.6 antibodies to detect DNA:RNA hybrids in *E*. *coli rnhA*, *topA* and *topA topB* null mutants. Wild-type (RFM443) and *rnhA*::*cam* (MM84) cells were grown at 37°C to log phase, whereas *topA20*::Tn*10 ΔtopB gyrB*(Ts)/pSK760 (VU422), *topA20*::Tn*10 ΔtopB gyrB*(Ts)/pSK762c (VU425) and *topA20*::Tn*10 gyrB*(Ts) (RFM480) cells were grown at 30°C to log phase. RFM480 downshift is *topA20*::Tn*10 gyrB*(Ts) cells grown at 37°C to log phase and transferred for 45 min. at 30°C. Genomic DNA was extracted and dot blotting was performed as described in Materials and Methods. RNase HI–and + mean that the genomic DNA samples were respectively not treated and treated with RNase HI as described in Materials and Methods.

Because cSDR is strongly activated in cells lacking type 1A topos, it should be possible to detect R-loops in these cells. To test this, we performed dot blots with S9.6 antibody that recognizes DNA:RNA hybrids. S9.6 has been widely-used to detect and map R-loops in eukaryotic cells [41]. To validate this approach for *E*. *coli* cells, we considered two situations in which R-loop formation/accumulation has been supported by much experimental evidence and/or can be predicted to occur. The first situation is related to *rnhA* null mutants. Because of the absence of RNase HI and the occurrence of cSDR, R-loops are expected to accumulate in *rnhA* null mutants. To test that, genomic DNA was extracted from both the *rnhA* null mutant (MM84) and the isogenic wild-type strain (RFM443). Fig 5 demonstrates the accumulation of R-loops in the *rnhA* null mutant but not in the wild-type strain as expected (compare RFM443 and MM84,—and + RNase HI).

The second situation concerns *topA* null mutants. R-loop formation on plasmid, based on RNase HI-sensitive gel retardation and hypernegative supercoiling, has been shown to occur both *in vitro* and *in vivo*, in the absence of topo I but in the presence of gyrase [14, 51]. Based on these assays and on the growth inhibition and RNA degradation phenotypes that can be corrected by RNase HI overproduction, R-loop formation can be predicted to occur in our *topA* null mutant following a temperature downshift from 37°C to 30°C and below [17]. To test that, genomic DNA was extracted from our *topA* null mutant (RFM480: *topA20*:Tn*10*, *gyrB*(Ts)) 45 min after a temperature downshift from 37 to 30°C. Fig 5 shows the accumulation of R-loops in these cells as an RNase HI-sensitive spot could be detected in dot-blot experiments with S9.6 antibodies (RFM480 downshift, compare–and + RNase HI). Thus, these results establish the validity of the S9.6 antibodies to detect R-loops in *E*. *coli* cells and directly demonstrate, for the first time, the accumulation of R-loops in *topA* null and *rnhA* null mutants.

Next, we analyzed genomic DNA preps from three isogenic strains, RFM480 (*topA20*:Tn*10*, *gyrB*(Ts)), VU422 (*topA20*:Tn*10*, *gyrB*(Ts), *ΔtopB*/pSK760) and VU425 (*topA20*:Tn*10*, *gyrB*(Ts), *ΔtopB*/pSK762c) grown under the same conditions used for NGS and qPCR, i.e. 30°C up to an OD_600_ of 0.4. Fig 5 clearly shows the accumulation of R-loops in VU425 (pSK762c: no RNase HI overproduced) but not in VU422 (pSK760: RNase HI overproduced; very weak signal) or RFM480. Thus, under these conditions, growth at 30°C and no temperature changes, R-loops are only detected significantly when both type 1A topos are absent. Thus, the high level of cSDR replication correlates with an accumulation of R-loops in *topA topB* null cells.

### Amplification of a DNA region including *parC* and *parE* genes in *topA topB* mutants

Fig 3 also shows the presence of a DNA amplification in the *topA topB* mutant whether or not RNase HI was overproduced (top and middle panels, *amp*: from genomic position 2.99 to 3.24). As stated in the introduction, such an amplification that includes the *parC* (3.162) and *parE* (3.172) genes coding for the two subunits of Topo IV, is the most frequent compensatory mechanism for the absence of topo I [7]. DNA amplifications are very unstable as they are easily lost by a RecA-dependent mechanism [52]. There maintenance in a population of cells indicate that they confer a growth advantage. The other well-described compensatory mechanism is the occurrence of mutations reducing the supercoiling activity of gyrase (*gyrA* and *gyrB*). Normally, only one mechanism is sufficient to allow the growth of *topA* null mutants. We have previously shown that upon prolonged incubation on plates, our *topA* null *gyrB*(Ts) mutants, mostly those carrying the *topA20*::Tn*10* allele as compared to the *Δ(topA cysB)* allele, can generate larger colonies at a very high frequency [15]. These colonies are made of cells carrying a DNA amplification of the chromosomal region including *parC* and *parE*. This is likely because the *gyrB*(Ts) mutation is not a naturally selected one that arose to compensate for the absence of *topA*, and it is therefore probably not optimal for compensation. Thus, despite the fact that our *topA* null mutants can grow without the *parC parE* amplification (S2 Fig), as also shown in this work, when it occurs it confers a growth advantage. However, according to our results the situation might be different for the *topA topB* null mutant as in addition to the *gyrB*(Ts) mutation, it also carries an amplification of the *parC parE* region (Fig 3). This may suggest that excess negative DNA supercoiling is more harmful for *topA topB* null cells than it is for *topA* null cells and/or that more relaxation activity is required in the double mutant.

**Fig 6:**
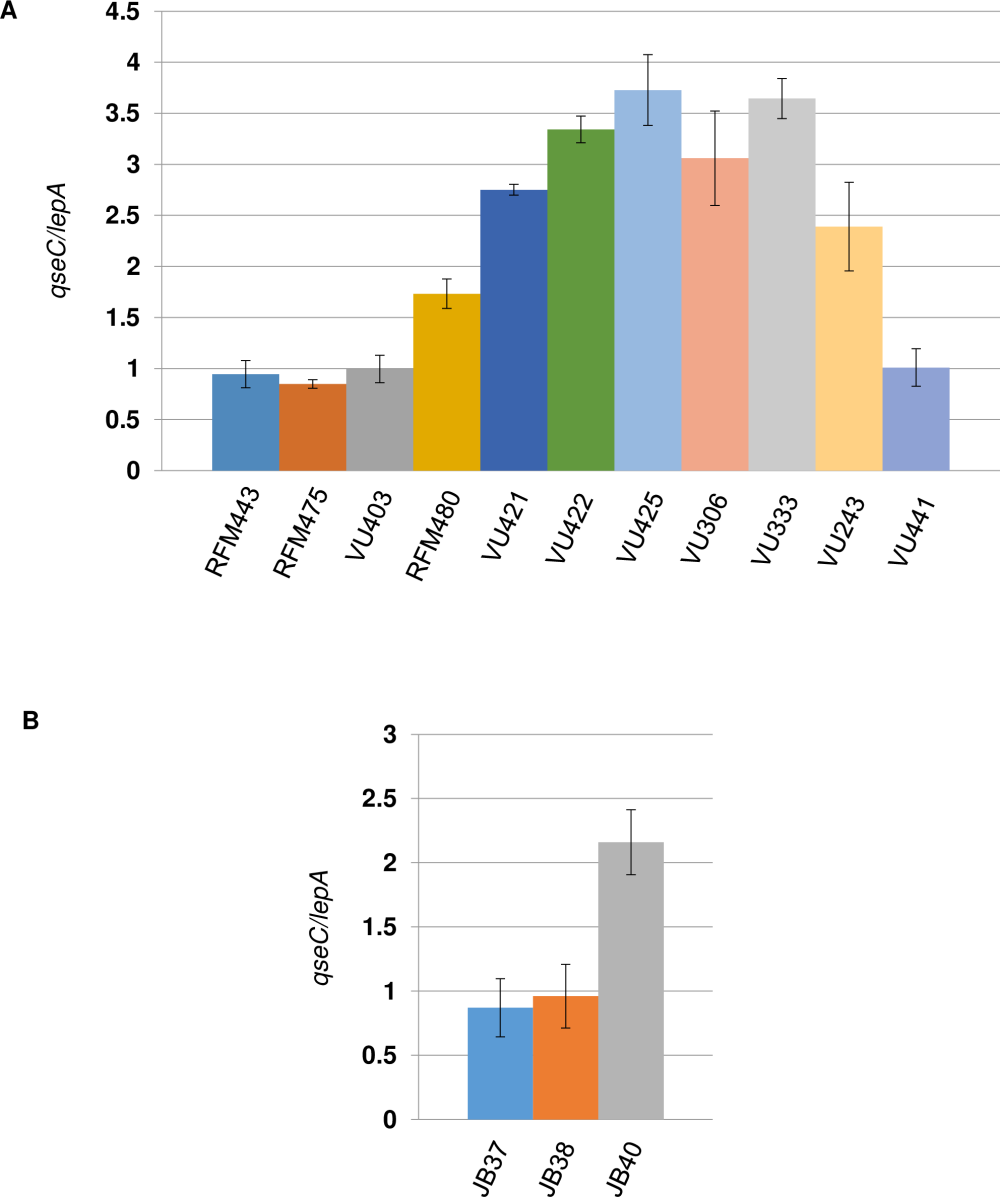
*qseC/lepA* ratio in *E*. *coli topA* and *topA topB* null mutants. In (a) wild-type (RFM443) cells were grown at 37°C to log phase, whereas *Δ(topA cysB) gyrB*(Ts) (RFM475), *ΔtopB761*::*kan gyrB*(Ts) (VU403), *topA20*::Tn*10 gyrB*(Ts) (RFM480), *topA20*::Tn*10 ΔtopB gyrB*(Ts) (VU421), *topA20*::Tn*10 ΔtopB gyrB*(Ts)/pSK760 (VU422), *topA20*::Tn*10 ΔtopB gyrB*(Ts)/pSK762c (VU425), *Δ(topA cysB) ΔtopB*::*kan gyrB*(Ts)/pSK760 (VU306), *Δ(topA cysB) ΔtopB*::*kan gyrB*(Ts)/pSK762c (VU333) *topA20*::Tn*10 ΔtopB dnaT18*::*aph gyrB*(Ts) (VU441) and *Δ(topA cysB) ΔtopB*::*kan gyrB*(Ts) *ΔrecA306 srlR301*::Tn*10* (VU243) were grown at 30°C to log phase. Genomic DNA was extracted and qPCR was performed as described in Materials and Methods. In (b) *ΔtopB*::*kan* transductants of SS12 (*Δ(topA cysB) gyrB*(Ts)/pET11-*parEC*; strains JB37 and JB38)) and RFM475 (*Δ(topA cysB) gyrB*(Ts); strain JB40) were grown at 30°C to log phase and genomic DNA was extracted and qPCR was performed as described in Materials and Methods.

We used qPCR to look for a DNA amplification of the *parC parE* genomic region in various isogenic strains used in the present study. Cells were grown and DNA extracted as done for MFA by NGS, i.e. 30°C up to an OD_600_ of 0.4, except that the spectinomycin treatment was omitted. For the qPCR, we used probes that correspond to *qseC* (genomic position 3.169), located between *parC* and *parE*, and *lepA* (genomic position 2.705). Fig 6A shows that the *qseC/lepA* ratio is close to one for both the wild-type (RFM443; 0.95) and the *Δ(topA cysB) gyrB*(Ts) (RFM475; 0.85) strains. In the absence of DNA amplification, the ratio is expected to be near one as *qseC* and *lepA* are close to each other, and therefore no significant copy number variations related to replication from *oriC* are expected to be seen. The result for the *topA* null strain confirms the absence of the *parC parE* amplification when *topB* is present as shown by NGS for the strain carrying the *Δ(topA cysB)* allele (S2 Fig; RFM475). Furthermore, the reverse is also true, i.e. no amplification is seen in the absence of *topB* when *topA* is present (Fig 6A, VU403). When the qPCR was performed with the *topA* null mutant carrying the *topA20*::Tn*10* allele (Fig 6A; RFM480), a small but reproducible DNA amplification of the *parC parE* region (*qseC/lepA* ratio of 1.7) was observed. So, the selective pressure to keep the *parC parE* amplification is stronger when the cells carry the *topA20*::Tn*10* allele as compared to the *Δ(topA cysB)* allele. These results are in agreement with our previous observations showing that the phenotypes of *topA* null and *topA topB* null mutants are stronger when they carry the *topA20*::Tn*10* allele instead of the *Δ(topA cysB)* one [33].

Next, we analyzed two pairs of *topA topB* null strains that have been constructed in our laboratory. Each pair includes strains carrying the *gyrB*(Ts) allele and either pSK760 (RNase HI overproduction) or pSK762c (control, no RNase HI overproduction). The first pair, VU422 (pSK760) and VU425 (pSK762c), the one that has been used in MFA by NGS, includes strains with the *topA20*::Tn*10* mutation and the *ΔtopB* allele from the Keio collection [53]. The second pair, VU306 (pSK760) and VU333 (pSK762c) includes strains with the *Δ(topA cysB)* mutation and the *ΔtopB*::*kan* allele from ref. [30]. For the first pair, the *topB* null allele was introduced before the *topA* null allele, whereas for the second one, the *topB* null allele was introduced after the *topA* null allele. Fig 6A clearly shows the presence of a DNA amplification of the *parC parE* region in all the strains (*qseC/lepA* ratios from 3.0 to 3.7; the ratio is 2.8 for strain VU421, the *topA topB* null mutant from which VU422 and VU425 were obtained). So the *parC parE* amplification is observed whether RNase HI is overproduced or not, as shown for the NGS results (Fig 3) and irrespective of the *topA* and *topB* null alleles present in the strains.

We also looked for *parC parE* amplifications in *topA topB* null strains carrying the *recA* (VU243) or *dnaT* (VU441) mutation that were shown respectively to fully inhibit or considerably reduce cSDR (Fig 4). Fig 6A shows the presence of a DNA amplification in the *topA topB* null strain carrying the *recA* mutation, as the *qseC/lepA* ratio is close to 2.5. Although this result may suggest that deleting *recA* did not eliminate the need for topo IV overproduction, it is difficult to interpret as the loss of duplications/amplifications is a RecA-dependent process [52]. Conceivably, introducing the *recA* mutation could have stabilized a *parC parE* amplification that was already present in the *topA topB* null mutant. Fig 6A shows the absence of a *parC parE* duplication/amplification in the *topA topB* null strain carrying the *dnaT* mutation, as the *qseC/lepA* ratio is close to one. This result shows that the *dnaT* mutation not only inhibited cSDR as shown above, but also allowed the *topA topB* null mutant to grow despite the lack of a *parC parE* amplification. This supports the hypothesis that the major problem of cells lacking type 1A topos activity is related to R-loop-dependent replication initiated from the PriA-dependent primosome that also includes DnaT.

### The presence of a plasmid from which topo IV can be overproduced can bypass the need to maintain the *parC parE* amplification in *topA topB* null mutants

If the amplification of a genomic DNA region including *parC* and *parE* genes is indeed required to allow topo IV overproduction, the need for this amplification should be bypassed by introducing a plasmid from which topo IV can be produced into *topA topB* strains. To test this, we used the plasmid pET11-*parEC* producing a ParEC fusion protein that was shown to be active as a topo IV both *in vitro* and *in vivo* [54], and that could complement the growth defect of the *topA* null strain RFM475 [32]. This plasmid was first introduced into the *topA gyrB*(Ts) strain (RFM475) and then transduction with P1*vir* was performed to introduce the *ΔtopB*::*kan* allele into RFM475/pET11-parEC. The transduction was also performed in parallel in the RFM475 strain carrying no plasmid. The *ΔtopB*::*kan* transductants appeared after 24 and 48 hours of incubation respectively for the RFM475 strain with and without pET11-*parEC*. Upon re-streaking them at 37°C, the RFM475 transductants carrying pET11-*parEC* grew slightly better than the ones of strain RFM475 carrying no plasmid. At 30°C, no significant differences in term of colony number and size could be seen between these transductants. Fig 6B shows *qseC/lepA* ratios close to 1 (0.87 and 0.96) and above 2 (2.16) for *ΔtopB*::*kan* transductants of RFM475 respectively carrying (2 clones; JB37, JB38) or not carrying (1 clone; JB40) pET11-*parEC*. This is the expected result if the selective pressure to maintain the amplification of a genomic region including *parC* and *parE* is related to topo IV overproduction. Thus, the amplification of a genomic DNA region including *parC* and *parE* allows *topA topB* mutants to grow because of topo IV overproduction.

### Plasmid DNA is more relaxed in *topA topB* null cells as compared to *topA* null cells

The *parC parE* amplification is likely maintained in *topA topB* null cells to provide a higher level of DNA relaxation activity, via topo IV, possibly to limit the accumulation of R-loops and its associated unregulated replication. We used plasmids pACYC184ΔEN and pACYC184ΔHE as markers for the global supercoiling level in isogenic *topA* and *topA topB* null strains. Various portions of the *tet* gene have been deleted in these plasmids so that they can be used to evaluate the global supercoiling level, on which topo IV would mostly act. Topo I acts on local transcription-induced supercoiling that is generated by *tet* transcription on pACYC184 [55, 56]. S4 Fig clearly shows that more plasmid topoisomers migrated toward the relaxed state in the *topA topB* null strain (CT170) as compared to the *topA* null strain (RFM475). This supports the hypothesis that indeed topo IV overproduction provides more DNA relaxation activity to *topA topB* null cells.

## Discussion

Of all topoisomerases, enzymes of the type1A family are the most ancient and the only ubiquitous [2, 3]. In fact, type 1A topos are among the few proteins involved in DNA metabolism that are found in nearly all organisms of the three domains of life [57]. Because of the variety of substrates that these enzymes can use for activity, it has been difficult to identify their main cellular functions. The recent finding that type 1A topos of the two subfamilies, topo I and III, have RNA topo activity may further complicate the picture [58]. Nevertheless, this observation supports the hypothesis that type 1A topos were present in the RNA world and therefore in LUCA (Last Universal Common Ancestor) for the three domains of life. Moreover, this finding together with the phylogeny and the known activities of type 1A topos, suggest that gene duplication(s) that led to topo I and III occurred in LUCA [3].

In this context, the first important finding reported here that both *E*. *coli* topo I and III control R-loop formation/accumulation, may suggest that R-loops have been a problem early in the evolution of life, and that regulating their formation is an important function for type 1A topos, especially to inhibit unregulated replication. Whether or not an RNA topo activity is involved in the inhibition of R-loop formation is possible and need to be investigated. The second important finding reported in this work, is the observation that excess R-loop-dependent replication in the chromosomal Ter region appears to be largely responsible for the “classical” phenotype of *topA topB* null mutants that includes the formation of long filaments and the accumulation of unsegregated DNA. Thus unregulated replication can be considered as a major mechanism by which R-loops could threaten genome integrity. The third important finding concerns the viability of type 1A topos that can now be explained by the presence of a DNA amplification allowing topo IV to be overproduced. This suggests that the major role of type 1A topos in bacteria is to control the level of negative supercoiling. In fact, in bacteria that only have one type 1A enzyme, topo I, the best type 1A enzyme to relax DNA, not topo III, is present. Furthermore, in many of them, topo IV is also absent which could explain why in that case topo I is required for viability [59].

### Topo I and III in R-loop formation and cSDR

Previous results of *in vitro* experiments have suggested that *E*. *coli* type 1A topos can act at least at two levels to prevent the accumulation of R-loops. They can prevent their formation by relaxing transcription-induced supercoiling and they can likely destabilize R-loops by using them as hot-spots for DNA relaxation activity [14, 29, 60]. This activity may be reminiscent of a recently described reaction called D-loop dissolution [61]. In this reaction, yeast Top3 was able to dissolve Rad51 (RecA ortholog)-mediated D-loops on supercoiled templates, a reaction that was also accompanied by the simultaneous relaxation of the DNA template. *In vitro*, as predicted from the known biochemical properties of the two *E*. *coli* type 1A topos, an R-looped DNA template was shown to be a relatively better substrate for topo III than topo I, whereas the reverse situation was observed for a transcribed DNA template [29].

The results presented here showed that deleting *topB* from *topA* null mutants significantly stimulated both cSDR and the accumulation of R-loops detected by the S9.6 antibodies. Thus, by inhibiting the accumulation of R-loops, topo III can prevent unregulated replication. However, this function of topo III is only seen when topo I is absent. This suggests that topo III acts after topo I to prevent the accumulation of R-loops. Indeed, because topo III, unlike topo I, is a protein of low abundance, it is unlikely to act during transcription. In fact, a significant effect of topo III on transcription-induced negative supercoiling in a *topA* null mutant, was only observed when it was overproduced [29]. It is therefore more probable that topo III acts on the R-loop to destabilize it. This activity would be more compatible with its high specificity for ssDNA. Thus, in agreement with their biochemical properties and as shown *in vitro*, topo I, by interacting with RNAP, would act preferentially to relax transcription-induced supercoiling, whereas topo III would rather act on the R-loop to destabilize it.

The results presented in this work confirm the previous findings that a limited number of chromosomal locations can be used as *oriKs* in *rnhA* null cells [37, 42, 43]. Moreover, despite the fact that deleting *topA* can lead to non-sequence-specific R-loop formation via hypernegative supercoiling [17], *oriK* activity was observed almost exclusively at one location on the chromosome of our *topA* null mutant. Furthermore this location corresponded to an *oriK* originally identified in *rnhA* null mutants, the Ter located one. These results strongly suggest that the majority of R-loops do not lead to PriA-dependent replication. Factors such as the frequency of R-loop formation, the stability of the R-loops and some unknown additional properties of the R-loops and/or the surrounding nucleotide sequence are most likely very important for *oriK* activity. In this context, we can predict the occurrence of competing activities between proteins of low abundance such as PriA and topo III for the stable R-loops that have escaped degradation by RNase HI in the *topA* null mutant. Therefore, topo III would be specifically targeted to stable R-loops that can also be used by the PriA-dependent primosome, a function that would be compatible with its low copy number in the cell. In this context, it is important to mention that topo III, PriA and RNase HI have been shown to interact with SSB, the first protein that is predicted to bind to the ssDNA portion of an R-loop *in vivo* [62–65].

Despite the fact that the level of cSDR was shown to be higher in *rnhA* mutants that in *topA topB* null mutants, only in the later that the strong pathological state was observed [33, 34]. Here, our results of MFA by NGS revealed that the Ter located peak that likely correspond to an *oriK* site, was much higher in *topA topB* null cells as compared to *rnhA* null cells. It is therefore reasonable to propose that the pathology of cells lacking type 1A topos is related, at least partially, to this strongly activated origin of replication. The question is then how the high level of replication initiation in the Ter region can contribute to this pathological state. We believe that it could be related to hyper-recombination. Indeed, the chromosomal Ter region is known to be hyper-recombinogenic [66–68] and DSBs (double-strand breaks) are found in this region [69]. DSBs are substrates for the binding of the RecBCD complex that degrade the DNA up to a *chi* site, from which RecA proteins are loaded on the DNA to initiate homologous recombination. It has been shown that a *Ter*-blocked replication fork (*Ter*/Tus) can lead to replication fork collapse, i.e. the formation of a double-stranded end when a second replication fork moving in the same direction run into the *Ter*-blocked fork [70]. A high level of RecA and RecBCD-dependent recombination that requires the presence of Tus protein and *chi* sites, thus likely related to forks collapse, has been shown to occur at *TerA*, *B* and *C* sites in *rnhA* null but not in wild-type cells [68]. This was proposed to be due to cSDR initiated in the Ter region of *rnhA* null cells [68]. Considering the very high level of cSDR in the Ter region of *topA topB* null cells with replication forks arrested at *TerA* and *B* sites, forks collapse is expected to be very frequent and may lead to hyper-recombination in these cells. Interestingly, *topA* null mutants deleted for *recB* were previously shown to be barely viable [33]. The high level of hyper-recombination in the Ter region of *topA topB* null mutants could impede chromosome segregation, either because of the accumulation of Holliday junctions or because of the additional PriA-dependent replication initiated from D-loops that have been assembled by RecA during DSB repair.

We cannot exclude the possibility that a type 1A topo activity is required during cSDR to solve the topological problems of head-on collisions between replisomes or between a replisome and a heavily transcribed gene or operon, as we recently proposed [34, 47]. Such conflicts may threaten cell viability [43, 46]. Clearly more work are still required to fully understand the pathological state of cells lacking type 1A topos.

### Interplay between type 1A topos and topo IV

The fact that topo IV needs to be overproduced for *topA topB* null mutants to survive despite the presence of a *gyrB*(Ts) compensatory mutation, likely indicates a major problem related to excess negative supercoiling in this strain. The observation that the *dnaT* mutation can both correct the cSDR phenotype and allow the requirement for a *parC parE* amplification to be bypassed in a *topA topB* null strain, may suggest that PriA-dependent replication from R-loops is the main problem related to excess supercoiling in strain lacking type 1A topos.

We have previously shown that upon a temperature downshift, *topA* null mutants carrying the *gyrB*(Ts) allele accumulated both hyper-negatively supercoiled DNA and truncated RNAs, and stopped growing [17]. After less than two hours, the accumulation of full-length functional RNAs and growth resumption coincided with the relaxation of hyper-negatively supercoiled DNA by topo IV [16, 17]. When RNase HI was overproduced hyper-negatively supercoiled DNA barely accumulated and was rapidly relaxed by topo IV and, as a result, growth was not inhibited. In agreement with the involvement of R-loops in this phenotype, we have shown here that during the transient growth arrest following a temperature downshift from 37 to 30°C, the *topA* null *gyrB*(Ts) mutant accumulated R-loops as detected by the S9.6 antibodies. As predicted, when this *topA* null mutant was growing at 30°C (OD_600_, 0.4; no temperature downshift), no R-loops were detected.

Based on *in vivo* and *in vitro* results, we have previously proposed a self-promoting cycle of R-loop formation whereby negative supercoiling promotes R-loop formation, which, in turn increases negative supercoiling following gyrase activity before the action of RNase HI [71]. This further increase R-loop formation. Ultimately, hypernegative supercoiling leads to extensive non-sequence specific R-loop formation [17]. In our model, RNase HI overproduction would efficiently compete with gyrase to prevent hypernegative supercoiling whereas topo IV would act later to relax this hypernegative supercoiling before its transcription. The involvement of topo IV in the inhibition of R-loop formation is also supported by our observation that *topA rnhA gyrB(Ts)* mutants carry an amplification of the *parC parE* region (*qseC/lepA* ratio slightly above 2).

Presumably, in the absence of both type 1A topos, R-loop formation/accumulation would be so efficient at least at some loci used to initiate cSDR that both RNase HI and topo IV would need to be overproduced to inhibit unregulated replication. It is also possible that the RNA of some R-loops, like the one used as a primer to initiate ColE1 replication, is resistant to RNase HI. In that case, the action of a topo could be required to prevent R-loop formation. Furthermore, at some specific sites R-loop formation may depend only on locally induced negative supercoiling during transcription. In this situation, only a type 1A topo would be able to inhibit their formation. Thus, our results suggest that in *E*. *coli*, topo I, III and IV all participate, to different extents, in the control of replication from R-loops to maintain the stability of the genome.

## Materials and methods

### Bacterial strains and plasmids

The bacterial strains used in this study are listed in S1 Table and are all derivatives of *E*. *coli* K12. S1 Table also gives the details on their constructions as well as the list of plasmids used in this study. Transductions with phage P1*vir* were done as described previously [32]. PCR with appropriate oligonucleotides were performed to confirm the transfer of the expected alleles in the selected transductants.

### Marker frequency analysis by next generation sequencing

Cells were grown overnight in liquid LB medium supplemented with the appropriate antibiotics. Overnight cultures were diluted in LB medium to obtain an OD_600_ of 0.01 and grown at the indicated temperature to on OD_600_ of 0.4. When indicated, spectinomycin (400μg/ml) was added and the cultures were incubated for an additional two hours at the same temperature. Samples of 10 ml were transferred in tubes filled with ice and the cells were recovered by centrifugation. Genomic DNA was extracted by using either the GenElute bacterial genomic DNA kit (Sigma Aldrich) or the QIAamp DNA mini kit (Qiagen). Similar results were obtained for both kits whether the genomic DNA was used for NGS, qPCR or dot-blots with S9.6. For the stationary phase wild-type (RFM443) cells, culture were incubated overnight and 1.5 ml samples were used for the genomic DNA extraction. The purity of the various DNA preps was evaluated by using the Nanodrop (Thermofisher) and the DNA concentrations were determined by using the Qubit dsDNA Assay kit (Invitrogen) with the Qubit Fluorometer (Thermofisher). Shotgun libraries with PCR were prepared for Illumina sequencing. Sequencing was performed by using Illumina HiSeq 2500 v4 (Génome Québec, Montréal, Canada) to determine sequence copy number. Bioinformatics analysis was performed at the Canadian Centre for Computational Genomic (C3G, McGill University, Montréal, Canada). For the read mapping (11 to 16 million sequencing reads per sample), the *E*. *coli* K12 W3310 genomic sequence AP009048.1 was used as the reference. To reduce miscalculation of depth of coverage due to reads mapping at multiple places in the genome, a minimum mapping quality of 10 (phred scale based) was used for a read to be kept during the calculation of depth of sequencing. In Figs 2 and 3, the number of reads were normalized against a spectinomycin-treated wild-type control to take into account differences in read depth across the genome of spectinomycin treated cells. Enrichment in 500 bp windows (on average) across the genome (10,000 points) was calculated and loess regression curves were generated with loess_span parameters set to 0.1.

### qPCR

Genomic DNA for qPCR was prepared as described above for NGS. The Quantinova SYBR Green PCR kit (Qiagen) was used with a Rotor-Gene 6000 (Corbett) apparatus. For each experiment and each set of primers two tubes were prepared that contained respectively 8 and 20 ng of genomic DNA. Experiments were repeated at least twice for each set of primers. The ratios were determined by using the 2^-Δct^ formula and standard deviations were calculated from these values. The primers were designed by using the PrimerQuest tool (IDT). Forward and reverse primer sequences (5’-3’) were GAGTACCGGGCAGACCTATAA and AGCCTACTTCGCCACATTTC for *lepA*, CGAGACTTCAGCGACAGTTAAG and CCTGCGGATATTTGCGATACA for *ydcM* and CTGGACTCACTGGATAACCTTC and TGCGCCGTGTGGTAAATA for *qseC*.

### Dot blots with S9.6 antibodies

Genomic DNA for the dot blots with S9.6 antibodies was prepared as described above for NGS except that the amount of RNase A added was reduced by half. For each genomic DNA prep, two tubes containing 300 ng of DNA in RNase III reaction buffer (Ambion) were prepared. In one tube RNase III (2U; Ambion/Invitrogen; a ribonuclease specific to dsRNAs) was added, whereas in the second tube RNase III and RNase HI (0.8 μg; from Kefei Yu, Michigan State University) were added. The tubes were incubated at 37°C for 3 hours and the DNA was purified by phenol/chloroform extraction and EtOH precipitation. The DNA was resuspended in 20 μl of TE. The RNase III treatment was found to be necessary as the S9.6 antibody can also recognize RNA:RNA hybrids (dsRNAs), albeit with a 5 to 6 fold lower affinity than DNA:RNA hybrids [72]. Moreover, a strong RNase H-resistant signal in dot blots, especially for the genomic DNA from the *topA topB* null mutant not overproducing RNase HI, was found to be RNase III-sensitive. We believe that this signal might be due to the accumulation of short truncated RNAs, especially highly structured rRNA fragments, due to R-loop formation in *topA* null mutants as shown previously [73, 74]. In fact, S9.6 was recently shown to be able to immuno-precipitate dsRNAs from yeast cells, and a RNase III treatment was found to be required to generate an accurate map of R-loops in the genome of yeast cells [75].

Dot blotting was performed essentially as described previously [76, 77]. Ten μl of DNA were spotted on a Hybond-N^+^ membrane (Amersham). The membrane was UV-crosslinked (UV Stratalinker 1800), blocked in 5% milk in TBST and incubated overnight at 4°C with 30 μg of S9.6 antibody (obtained from Dr Michael Wilson, University of Toronto, Canada). The membrane was washed 3 times in TBST and the secondary antibody (1:500; Stabilized Goat Anti-Mouse IgG HRP, Thermo scientific) was added for 1 hour at room temperature. The HRP signal was revealed by using the SuperSignal West Pico PLUS kit (Thermo scientific) and the membrane was exposed to an autoradiography film.

## Supporting information

S1 Fig qRT-PCR to determine the relative expression level of *dnaT* in the *dnaT18*::*aph* mutant (wild-type/*dnaT18*::*aph* ratio). Wild-type (RFM443) and *dnaT18*::*aph* (JB02) cells were grown overnight at 37°C and diluted to on OD_600_ of 0.01 in fresh LB medium. Cells were grown to log phase (OD_600_ of 0.4) at the same temperature and RNA was extracted by using the RNAprotect Bacteria Reagent (Qiagen) and the RNeasy Mini kits (Qiagen). The RNA preps were then treated with DNase (TURBO DNA-free kit from Invitrogen). The purity and the concentration of the various RNA preps were evaluated by using the Nanodrop (Thermofisher). The QuantiNova SYBR Green RT-PCR kit (Qiagen) with the Rotor-Gene 6000 (Corbett) apparatus were used for the qRT-PCR experiments. For each experiment and each set of primers 3 tubes were prepared that contained respectively 2, 20 and 100 ng of RNA. Experiments were repeated at least twice for each set of primers. The WT/*dnaT18*::*aph* ratios were determined by using the 2^-Δct^ formula and standard deviations were calculated from these values. The primers were designed by using the PrimerQuest tool (IDT). Forward and reverse primer sequences (5’-3’) were CATGTGCAGTGGCAACAAA and CAGGTTCGCTGACCGTATT for *dnaT*, CATCACCGTGGCCGATATTA and TCGATCACCAGCAGATCAAC for *dnaC* and GAGCAAGGCTATCTGACCTATG and GCCCATGTCGTTGATCATTTG for *rpoD*.
(TIF)

S2 Fig Replication profile of the *Δ(topA cysB) gyrB*(Ts) RFM475 strain. *Δ(topA cysB) gyrB*(Ts) (RFM475) cells were grown at 35°C to log phase and treated with spectinomycin, and genomic DNA was extracted for NGS as described in Materials and Methods. The read counts (Log2) normalized against a wild-type (RFM443) spectinomycin treated control were plotted against chromosomal coordinates (W3110). The gray line is the loess regression curve (see Materials and Methods). The green arrows on the top of the profiles point to potential cSDR origins (*oriKs*) and the blue ones at the bottom of the profiles point to *TerA* and *TerB* polar replication termination sequences.
(TIF)

S3 Fig Comparison of the NGS and qPCR methods to determine the *ydcM/lepA* ratio in *E*. *coli rnhA* and *topA topB* null mutants. Samples of genomic DNA preps used in the NGS experiments (Fig 1 to 3) from wild-type (RFM443), *rnhA*::*cam* (MM84), *rnhA*::*cam dnaT18*::*aph* (JB04), *topA20*::Tn*10 ΔtopB gyrB*(Ts)/pSK760 (VU422) and *topA20*::Tn*10 ΔtopB gyrB*(Ts)/pSK762c (VU425) strains were used in qPCR experiments to determine their respective *ydcM/lepA* ratio. The histogram shows for each strain the *ydcM/lepA* ratio as determined by qPCR (left) and NGS (right).
(TIF)

S4 Fig Plasmid DNA supercoiling in *topA* and *topA topB* null strains. Wild-type (RFM443), *gyrB*(Ts) (RFM445), *Δ(topA cysB) gyrB*(Ts) (RFM475) and *Δ(topA cysB) ΔtopB*::*kan gyrB*(Ts) (CT170) cells carrying either pACYC184ΔEN or pACYC184ΔHE were grown at 30°C to an OD_600_ of 0.4. The cells were transferred in a tube filled with ice and recovered by centrifugation. Plasmid DNA was extracted by using the Monarch plasmid miniprep kit (NEB) and electrophoresis was performed in 0.7% agarose gel in 0.5 x TBE buffer containing 7.5 μg/ml of chloroquine. The gel was stained with SYBR Gold (Thermofisher) and photographed under UV light. Under this chloroquine concentration, the relaxed topoisomers (rel) migrate faster than the negatively supercoiled (sup) topoisomers (Usongo V, Nolent F, Sanscartier P, Tanguay C, Broccoli S, *et al*. (2008) Depletion of RNase HI activity in *Escherichia coli* lacking DNA topoisomerase I leads to defects in DNA supercoiling and segregation. Mol Microbiol 69:968–981; Mutations reducing replication from R-loops suppress the defects of growth, chromosome segregation and DNA supercoiling in cells lacking topoisomerase I and RNase HI activity (2016) Usongo V, Martel M, Balleydier A and Drolet M. DNA Repair (Amst) 40:1–17.). The red dots point to the topoisomers bands that correspond to the mean superhelical density of the plasmid DNA in the various samples. pACYC184ΔEN and pACYC184ΔHE were constructed by deleting respectively the EcoRV-NruI and HindIII-EcoRV DNA fragments of pACYC184. These deletions inactivated the *tet* gene of pACYC184.
(TIF)

S1 Table *Escherichia coli* strains and plasmids used in this work. The strains were constructed as described in Material and Methods.
(DOC)

S1 Numerical data *ydcM/lepA* and *qseC/lepA* ratios determined by using the 2^-Δct^ formula as described in Material and Methods. Means and standard deviations calculated from these values are also shown.
(XLSX)
