# Supplementary figures and images for: Topoisomerases I and III inhibit R-loop formation to prevent unregulated replication in the chromosomal Ter region of Escherichia coli
Source: PLoS Genet. 2018 Sep 17;14(9):e1007668. doi: 10.1371/journal.pgen.1007668 (PMC6160223; doi:10.1371/journal.pgen.1007668)

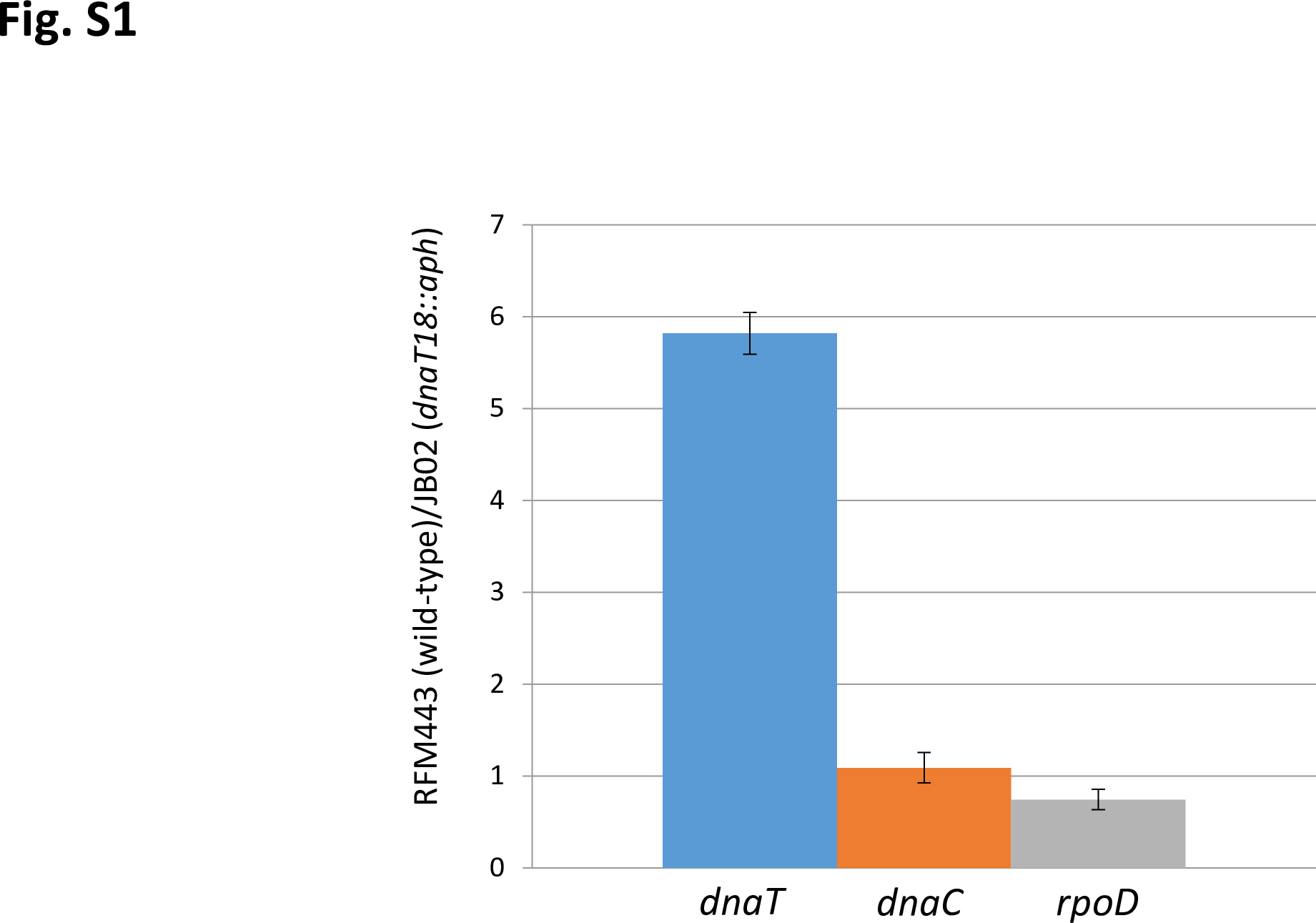

Supplement: S1 Fig — Wild-type (RFM443) and dnaT18::aph (JB02) cells were grown overnight at 37°C and diluted to on OD600 of 0.01 in fresh LB medium. Cells were grown to log phase (OD600 of 0.4) at the same temperature and RNA was extracted by using the RNAprotect Bacteria Reagent (Qiagen) and the RNeasy Mini kits (Qiagen). The RNA preps were then treated with DNase (TURBO DNA-free kit from Invitrogen). The purity and the concentration of the various RNA preps were evaluated by using the Nanodrop (Thermofisher). The QuantiNova SYBR Green RT-PCR kit (Qiagen) with the Rotor-Gene 6000 (Corbett) apparatus were used for the qRT-PCR experiments. For each experiment and each set of primers 3 tubes were prepared that contained respectively 2, 20 and 100 ng of RNA. Experiments were repeated at least twice for each set of primers. The WT/dnaT18::aph ratios were determined by using the 2-Δct formula and standard deviations were calculated from these values. The primers were designed by using the PrimerQuest tool (IDT). Forward and reverse primer sequences (5’-3’) were CATGTGCAGTGGCAACAAA and CAGGTTCGCTGACCGTATT for dnaT, CATCACCGTGGCCGATATTA and TCGATCACCAGCAGATCAAC for dnaC and GAGCAAGGCTATCTGACCTATG and GCCCATGTCGTTGATCATTTG for rpoD. (TIF) [file pgen.1007668.s001.tif]

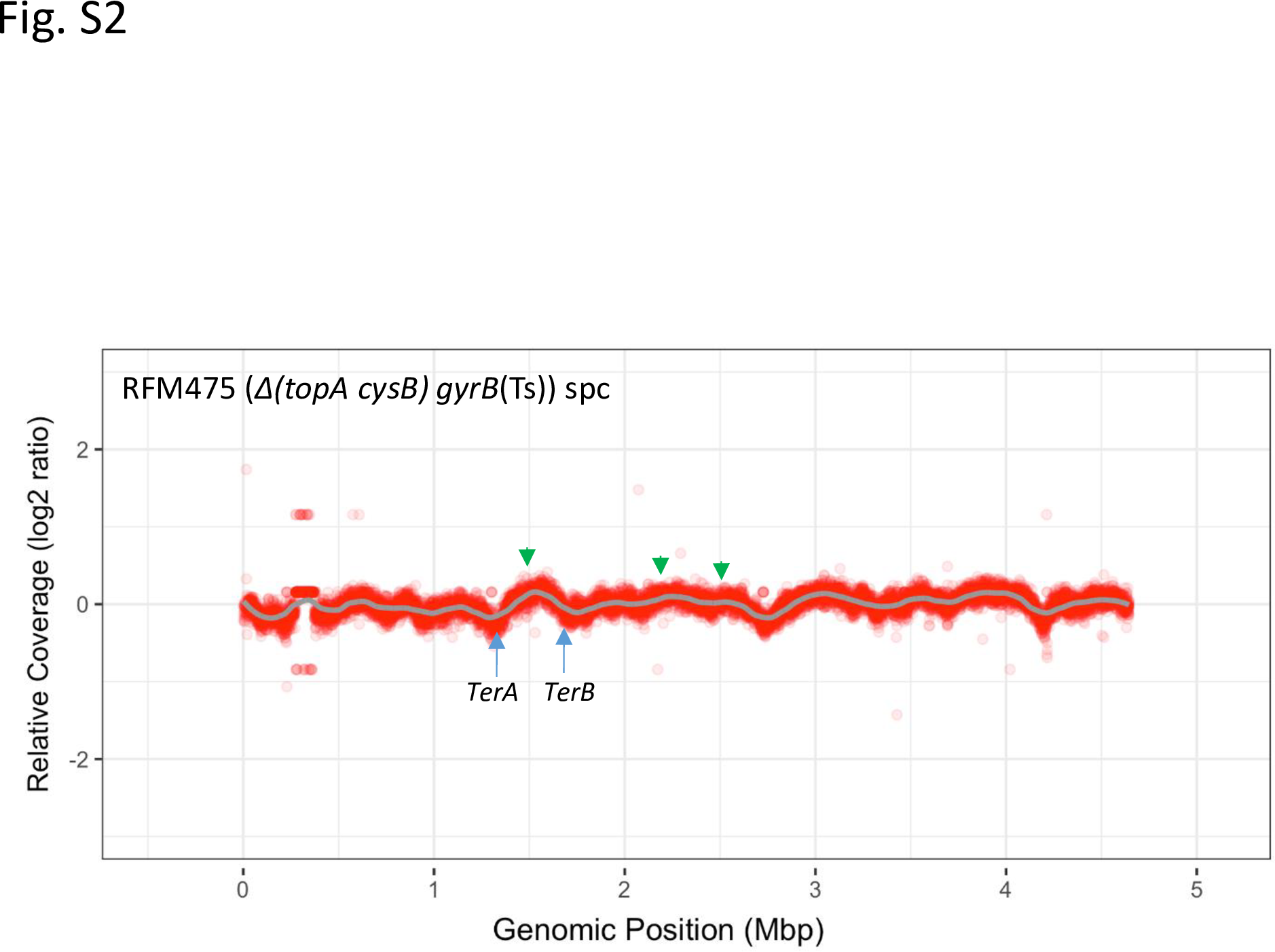

Supplement: S2 Fig — Δ(topA cysB) gyrB(Ts) (RFM475) cells were grown at 35°C to log phase and treated with spectinomycin, and genomic DNA was extracted for NGS as described in Materials and Methods. The read counts (Log2) normalized against a wild-type (RFM443) spectinomycin treated control were plotted against chromosomal coordinates (W3110). The gray line is the loess regression curve (see Materials and Methods). The green arrows on the top of the profiles point to potential cSDR origins (oriKs) and the blue ones at the bottom of the profiles point to TerA and TerB polar replication termination sequences. (TIF) [file pgen.1007668.s002.tif]

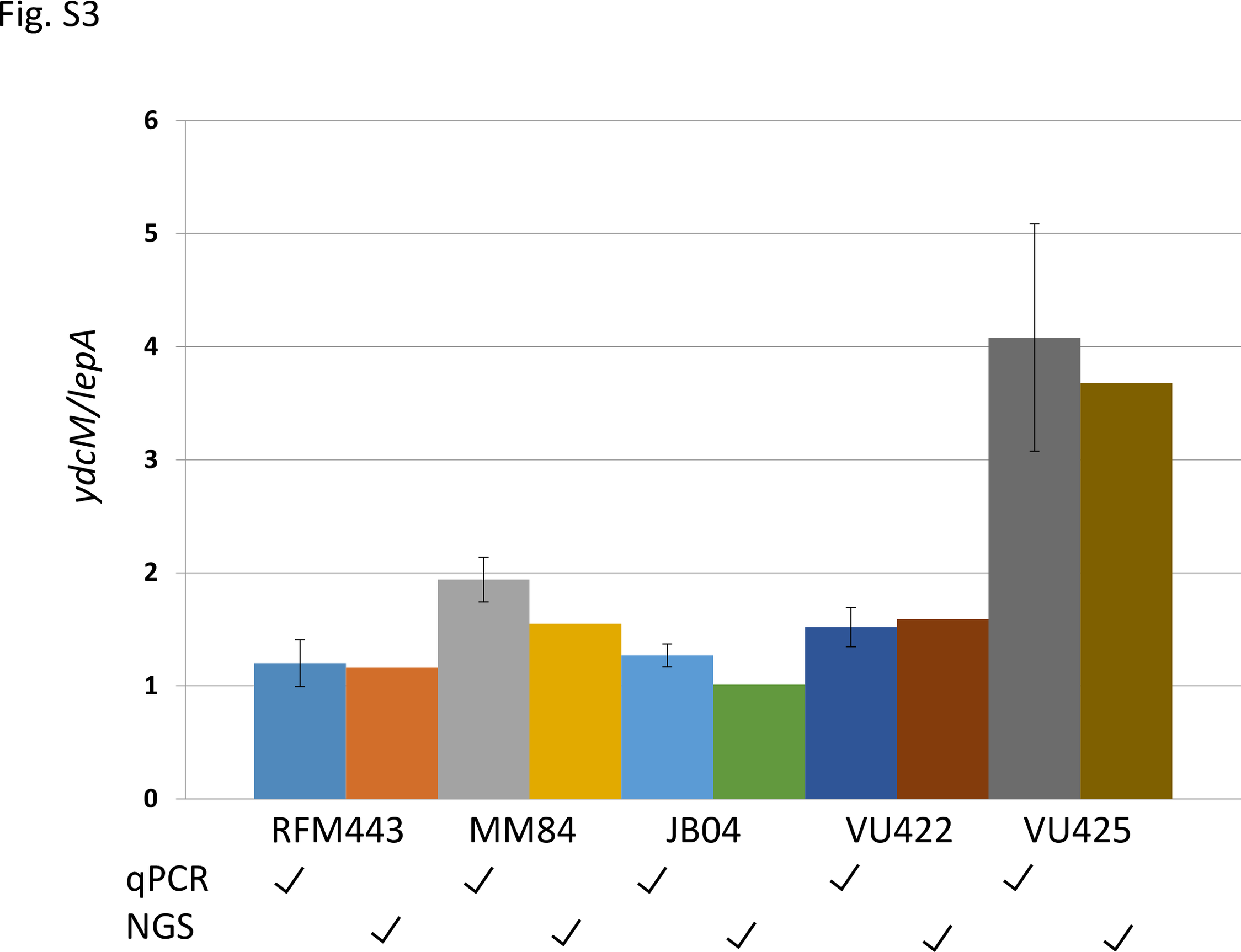

Supplement: S3 Fig — Samples of genomic DNA preps used in the NGS experiments (Fig 1 to 3) from wild-type (RFM443), rnhA::cam (MM84), rnhA::cam dnaT18::aph (JB04), topA20::Tn10 ΔtopB gyrB(Ts)/pSK760 (VU422) and topA20::Tn10 ΔtopB gyrB(Ts)/pSK762c (VU425) strains were used in qPCR experiments to determine their respective ydcM/lepA ratio. The histogram shows for each strain the ydcM/lepA ratio as determined by qPCR (left) and NGS (right). (TIF) [file pgen.1007668.s003.tif]

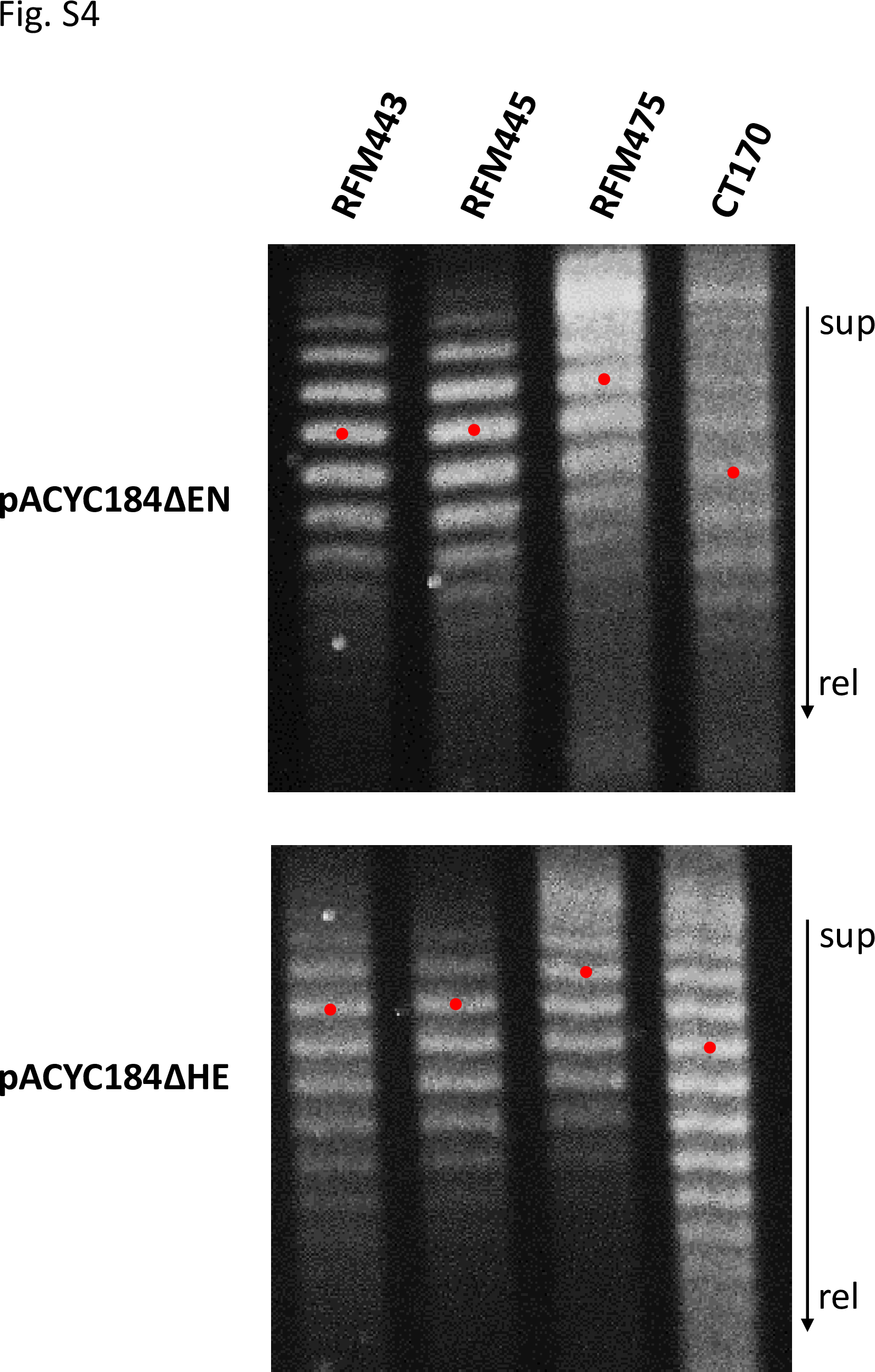

Supplement: S4 Fig — Wild-type (RFM443), gyrB(Ts) (RFM445), Δ(topA cysB) gyrB(Ts) (RFM475) and Δ(topA cysB) ΔtopB::kan gyrB(Ts) (CT170) cells carrying either pACYC184ΔEN or pACYC184ΔHE were grown at 30°C to an OD600 of 0.4. The cells were transferred in a tube filled with ice and recovered by centrifugation. Plasmid DNA was extracted by using the Monarch plasmid miniprep kit (NEB) and electrophoresis was performed in 0.7% agarose gel in 0.5 x TBE buffer containing 7.5 μg/ml of chloroquine. The gel was stained with SYBR Gold (Thermofisher) and photographed under UV light. Under this chloroquine concentration, the relaxed topoisomers (rel) migrate faster than the negatively supercoiled (sup) topoisomers (Usongo V, Nolent F, Sanscartier P, Tanguay C, Broccoli S, et al. (2008) Depletion of RNase HI activity in Escherichia coli lacking DNA topoisomerase I leads to defects in DNA supercoiling and segregation. Mol Microbiol 69:968–981; Mutations reducing replication from R-loops suppress the defects of growth, chromosome segregation and DNA supercoiling in cells lacking topoisomerase I and RNase HI activity (2016) Usongo V, Martel M, Balleydier A and Drolet M. DNA Repair (Amst) 40:1–17.). The red dots point to the topoisomers bands that correspond to the mean superhelical density of the plasmid DNA in the various samples. pACYC184ΔEN and pACYC184ΔHE were constructed by deleting respectively the EcoRV-NruI and HindIII-EcoRV DNA fragments of pACYC184. These deletions inactivated the tet gene of pACYC184. (TIF) [file pgen.1007668.s004.tif]
