# Supplementary material for: Topoisomerases I and III inhibit R-loop formation to prevent unregulated replication in the chromosomal Ter region of Escherichia coli
Source: PLoS Genet. 2018 Sep 17;14(9):e1007668. doi: 10.1371/journal.pgen.1007668 (PMC6160223; doi:10.1371/journal.pgen.1007668)
Supplement: S1 Table — The strains were constructed as described in Material and Methods. (DOC) [file pgen.1007668.s005.DOC]

**Table S1** *E. coli* strains and plasmids used.

| Name | Genotype or Relevant Genotype | Reference or Source |
| --- | --- | --- |
| AB004  CT77  CT170 | RFM443 *rnhA::cam fimD22::aph*  RFM443  *ΔtopB::kan*  RFM475 *ΔtopB::kan* | [1]  RFM443 x P1(MD897)  [2] |
| JB04  JB37  JB38  JB40  MD897  MM84  RFM443 | MM84 *dnaT18::aph*  SS12 *ΔtopB::kan*  SS12 *ΔtopB::kan*  RFM475 *ΔtopB::kan*  DM4100 *ΔtopB::kan*  RFM443 *rnhA::cam*  *Δ(codB-lacI)3 rpsL200 galK2*(Oc) *IN(rrnD-rrnE)1 rph-1* | MM84 x P1(VU186)  SS12 x P1(CT77)  SS12 x P1(CT77)  RFM475 x P1(CT77)  Lab collection  Lab collection  [3] |
| RFM445  RFM475  RFM480  SB265  SS12  VU186  VU243  VU294  VU296  VU306  VU333  VU403  VU421  VU422  VU425  VU441  pEM001  pEM003  pSK760  pSK762c | *Δ(codB-lacI)3 rpsL200 galK2*(Oc) *IN(rrnD-rrnE)1 rph-1 gyrB221* (Cou^r^) *gyrB203*(Ts)  *Δ(codB-lacI)3 rpsL200 galK2*(Oc) *IN(rrnD-rrnE)1 rph-1 gyrB221* (Cou^r^) *gyrB203*(Ts) *∆(topA cysB)204*  *Δ(codB-lacI)3, rpsL200, galK2*(Oc), *IN(rrnD-rrnE)1, rph-1 gyrB221* (Cou^r^) *gyrB203*(Ts) *topA20*::Tn*10*  RFM475 *ΔrecA306 srlR301*::Tn*10*  RFM475 pET11-*parEC*  RFM443 *dnaT18::aph*  CT170 *ΔrecA306 srlR301*::Tn*10*  RFM475 pSK760  RFM475 pSK762c  CT170 pSK760  CT170 pSK762c  RFM445 *ΔtopB761::kan*  RFM445 *ΔtopB* *topA20*::Tn*10*  VU421 pSK760  VU421 pSK762c  RFM445 *ΔtopB dnaT18::aph* *topA20*::Tn*10*  *rnhA* gene with its own promoter  like pEM001 but *rnhA* is mutated and  inactive  *rnhA* gene with its own promoter  like pSK760 but *rnhA* is mutated and  inactive | [3]  [3]  [3]  [4]  This work  This work  [4]  This work  This work  [4]  [4]  [4]  [4]  [4]  [4]  [4]  [5]  [5]  [3]  [3] |

pET11-*parEC* production of an active ParEC fusion [6]

protein

**Supplemental References**

1. Usongo V, Martel M, Balleydier A, Drolet M (2016) [Mutations reducing replication from R-loops suppress the defects of growth, chromosome segregation and DNA supercoiling in cells lacking topoisomerase I and RNase HI activity.](https://www.ncbi.nlm.nih.gov/pubmed/26947024) DNA Repair (Amst) 40:1-17.
2. Usongo V, Tanguay C, Nolent F, Egbe Bessong J, Drolet M (2013) Interplay between type 1A topoisomerases and gyrase in chromosome segregation in *Escherichia coli*. J Bacteriol 195:1758-1768.
3. Drolet M, Phoenix P, Menzel R, Massé E, Liu LF et al. (1995) Overexpression of RNase H partially complements the growth defect of an *Escherichia* *coli* delta *topA* mutant: R-loop formation is a major problem in the absence of DNA topoisomerase I. Proc Natl Acad Sci U S A 92:3526-3530.
4. Usongo V, Drolet M (2014) [Roles of type 1A topoisomerases in genome maintenance in *Escherichia coli*.](https://www.ncbi.nlm.nih.gov/pubmed/25102178) PLoS Genet 2014 Aug 7;10(8):e1004543.
5. [Massé E](https://www.ncbi.nlm.nih.gov/pubmed/?term=Massé E%5BAuthor%5D&cauthor=true&cauthor_uid=9139742), [Phoenix P](https://www.ncbi.nlm.nih.gov/pubmed/?term=Phoenix P%5BAuthor%5D&cauthor=true&cauthor_uid=9139742), [Drolet M](https://www.ncbi.nlm.nih.gov/pubmed/?term=Drolet M%5BAuthor%5D&cauthor=true&cauthor_uid=9139742) (1997) [DNA topoisomerases regulate R-loop formation during transcription of the *rrnB* operon in *Escherichia coli*.](https://www.ncbi.nlm.nih.gov/pubmed/9139742) *J Biol Chem* 272:12816-12823.
6. Lavasani LS, Hiasa H (2001) [A ParE-ParC fusion protein is a functional topoisomerase.](https://www.ncbi.nlm.nih.gov/pubmed/11456480) Biochemistry 40:8438-8443.
